# Engineering the production of conjugated fatty acids in *Arabidopsis thaliana* leaves

**DOI:** 10.1111/pbi.12695

**Published:** 2017-03-15

**Authors:** Olga Yurchenko, Jay M. Shockey, Satinder K. Gidda, Maxwell I. Silver, Kent D. Chapman, Robert T. Mullen, John M. Dyer

**Affiliations:** ^1^ USDA‐ARS US Arid‐Land Agricultural Research Center Maricopa AZ USA; ^2^ USDA‐ARS Southern Regional Research Center New Orleans LA USA; ^3^ Department of Molecular and Cellular Biology University of Guelph Guelph ON Canada; ^4^ Department of Biological Sciences University of North Texas Denton TX USA

**Keywords:** *Arabidopsis thaliana*, conjugated fatty acids, bio‐based feedstocks, biofuels, oil in leaves, *Vernicia fordii*

## Abstract

The seeds of many nondomesticated plant species synthesize oils containing high amounts of a single unusual fatty acid, many of which have potential usage in industry. Despite the identification of enzymes for unusual oxidized fatty acid synthesis, the production of these fatty acids in engineered seeds remains low and is often hampered by their inefficient exclusion from phospholipids. Recent studies have established the feasibility of increasing triacylglycerol content in plant leaves, which provides a novel approach for increasing energy density of biomass crops. Here, we determined whether the fatty acid composition of leaf oil could be engineered to accumulate unusual fatty acids. Eleostearic acid (ESA) is a conjugated fatty acid produced in seeds of the tung tree (*Vernicia fordii*) and has both industrial and nutritional end‐uses. *Arabidopsis thaliana* lines with elevated leaf oil were first generated by transforming wild‐type, *cgi‐58* or *pxa1* mutants (the latter two of which contain mutations disrupting fatty acid breakdown) with the diacylglycerol acyltransferases (*DGAT1* or *DGAT2*) and/or oleosin genes from tung. High‐leaf‐oil plant lines were then transformed with tung *FADX*, which encodes the fatty acid desaturase/conjugase responsible for ESA synthesis. Analysis of lipids in leaves revealed that ESA was efficiently excluded from phospholipids, and co‐expression of tung *FADX* and *DGAT2* promoted a synergistic increase in leaf oil content and ESA accumulation. Taken together, these results provide a new approach for increasing leaf oil content that is coupled with accumulation of unusual fatty acids. Implications for production of biofuels, bioproducts, and plant–pest interactions are discussed.

## Introduction

Modern society is heavily reliant on fossil oil as a source of fuel and chemical feedstocks, and with continued growth of world population, it is expected that this need will only continue to increase. Given the finite nature of fossil oils, as well as environmental concerns associated with its usage, there is a clear and pressing need to develop more sustainable, environmentally friendly alternatives to petroleum. The fatty acid components of plant oils are chemically similar to the long‐chain hydrocarbons of fossil oil and thus represent outstanding renewable sources of raw materials (Biermann *et al*., 2011; Carlsson *et al*., 2011; Dyer *et al*., 2008; Horn and Benning, 2016). Indeed, a significant proportion of oilseed crops is already diverted for usage as feedstocks for biodiesel production (Durrett *et al*., 2008), and government mandates for increased usage of renewable fuels have put additional pressure on agricultural production systems (Robbins, 2011; Zilberman *et al*., 2013). Given that oilseed crops also serve as important sources of food and feed, there is significant interest in developing novel approaches for producing high amounts of energy‐dense oils in dedicated, nonfood bioenergy crops and algae.

One approach that shows great potential for increasing oil production in plants is the elevation of neutral lipid content in vegetative biomass, such as leaves and stems (Chapman *et al*., 2013). While plant oils are typically derived from seeds, vegetative cell types also have the capacity to synthesize triacylglycerol (TAG), the major component of plant oil. In seeds, TAG accumulates to high levels (~35%–45% dry weight) and serves as an important carbon and energy reserve to fuel postgerminative growth, prior to photosynthetic establishment. In leaves, the TAG pool is much smaller (generally ≪1% dry weight) and more dynamic in nature, acting as a buffer against excess lipids and serving as a transient depot for fatty acids involved in membrane remodelling, lipid signalling, and/or fatty acid turnover (Chapman *et al*., 2012; Xu and Shanklin, 2016). However, recent research has shown that the TAG pool can be dramatically enhanced in vegetative cells using various engineering strategies that ‘push’ more carbon into the fatty acid biosynthetic pathway, ‘pull’ more fatty acids towards TAG synthesis, and ‘protect’ the TAG pool from turnover and/or fatty acid degradation (Vanhercke *et al*., 2013a; Weselake, 2016; Xu and Shanklin, 2016). While many of these studies have been conducted using the model plant *Arabidopsis thaliana*, combinatorial approaches have been used to increase oil content of tobacco leaves up to 30% dry weight (Vanhercke *et al*., 2016) and up to 4.7% in sugarcane (Zale *et al*., 2016), suggesting that commercial high oil biomass crops are just on the horizon.

The energy obtained from fossil oils can be derived from a number of alternative sources including wind, solar, nuclear, and hydropower. The petrochemical industry, however, requires carbon‐based feedstocks, and plant oils show great potential for fulfilling this need. Indeed, approximately 10% of plant oil is already used in various industrial applications (Biermann *et al*., 2011), but the fatty acid composition of vegetable oils is typically limited to just five basic fatty acid structures. There are hundreds of structurally diverse fatty acids synthesized in nature, and in many plant species, their seed oil is enriched in a single unusual fatty acid that can accumulate up to 90% of fatty acid composition (Badami and Patil, 1981; Smith, 1971). Many of the plants that produce these valuable oils, however, have poor agronomic traits or limited geographical growing areas. Therefore, a major goal of the plant biotechnology community has been to identify enzymes for unusual fatty acid synthesis and express them in higher yielding platform crops (Napier *et al*., 2014; Vanhercke *et al*., 2013b). Results to date have been mixed, however. For instance, engineering changes in fatty acid chain lengths or production of wax esters has been particularly successful (Lardizabal *et al*., 2000; Vanhercke *et al*., 2013b; Voelker *et al*., 1992), but production of unusual oxidized fatty acids has remained a challenge (Bates, 2016; Cahoon *et al*., 2007).

It is now widely recognized that many of the unusual fatty acids in plants are synthesized by divergent forms of fatty acid desaturase 2 (FAD2), an endoplasmic reticulum (ER) membrane‐bound enzyme that typically acts upon phosphatidylcholine (PC)‐linked oleate to produce linoleic acid (Okuley *et al*., 1994; Shanklin and Cahoon, 1998). Subtle changes in the polypeptide sequence of duplicated and diverged FAD2 enzymes alter their active site chemistry, allowing for production of a variety of oxidized products including hydroxy, epoxy, conjugated, and acetylenic fatty acids. Expression of diverged FAD2 enzymes in transgenic plants typically results in much lower accumulation of unusual fatty acids in seeds in comparison with seed oil from the plant in which the gene was sourced (Vanhercke *et al*., 2013b), and metabolic labelling studies have revealed inefficient removal of the unusual fatty acid from PC (Bates, 2016; Bates and Browse, 2011; Bates *et al*., 2014). This is particularly problematic for production of conjugated fatty acids, which can account for up to 25% of fatty acids in phospholipids of engineered seeds (Cahoon *et al*., 2006). Given that these fatty acids are likely disruptive to membrane structure, their accumulation in phospholipids is likely to contribute to the reported negative effects on embryo development and reduced germination potential (Cahoon *et al*., 1999, 2006).

Based on the recent success of increasing neutral lipid content in plant leaves, we asked whether this TAG pool might be engineered for accumulation of industrially important fatty acids, thereby bypassing some of the problems encountered with seeds. Towards that end, we focused on production of conjugated fatty acids, as these fatty acids have potential usage as industrial ‘drying oils’ in formulations of paints, inks, dyes, and resins (Sonntag, 1979), and they also have lipid‐lowering and possibly anticancer effects in animals (Lee *et al*., 2002; Thiel‐Cooper *et al*., 2001; Yuan *et al*., 2014). The source of genes for our study was the tung (*Vernicia fordii*) tree, which accumulates up to 80% eleostearic acid (ESA) in seed oil (Smith, 1971). The tung fatty acid conjugase (a diverged FAD2 termed FADX) has also previously been described, as have the tung diacylglycerol acyltransferases (*DGAT1* and *DGAT2*) and oleosin genes (Cao *et al*., 2014; Dyer *et al*., 2002; Shockey *et al*., 2006). Notably, prior studies revealed that tung DGAT2 likely plays a more important role in channelling of ESA into tung oil than DGAT1 (van Erp *et al*., 2015; Shockey *et al*., 2006).

Here, we developed a two‐step approach for producing high amounts of ESA in leaf tissues. The first step was to engineer elevated leaf oil content, in general, using strategies that were also likely to be important for accumulation of unusual fatty acids in leaves. Prior studies on ectopic expression of hydroxylases and conjugases in plants using constitutive gene promoters resulted in the accumulation of unusual fatty acids in plant seeds, but not in leaves (Iwabuchi *et al*., 2003; van de Loo *et al*., 1995). This suggested that leaf tissues contained robust mechanisms for exclusion of the unusual fatty acid from membranes, likely resulting in their degradation via peroxisomal β‐oxidation. To increase the possibility of channelling ESA to oil rather than turnover, we explored the usage of *Arabidopsis pxa1* mutant plants. PXA1 (peroxisomal ABC‐transporter 1) is a peroxisomal membrane protein that transports fatty acids into peroxisomes, and its disruption results in reduced fatty acid turnover and increase in leaf TAG (Kunz *et al*., 2009; Slocombe *et al*., 2009; Zolman *et al*., 2001). Given that disruption of *PXA1* blocks fatty acid breakdown, and accumulation of high amounts of ESA in leaf tissues might be toxic, we also explored the usage of *Arabidopsis cgi‐58* mutant plants. CGI‐58 (comparative gene identification‐58) is thought to act by stimulating the transport activity of PXA1, and loss of CGI‐58 also results in an elevation in leaf TAG (James *et al*., 2010; Park *et al*., 2013). To help redirect ESA from the fatty acyl‐CoA pool to TAG, we also explored the ectopic expression of the DGAT enzymes (tung DGAT1 and DGAT2) in leaf tissues, which has previously been shown to be an effective strategy for increasing oil content of plant leaves (Andrianov *et al*., 2010; Bouvier‐Navé *et al*., 2000; Vanhercke *et al*., 2014). We further combined the expression of tung *DGAT1* or *DGAT2* with expression of tung *OLEOSIN* (Cao *et al*., 2014), with the premise that oleosin can stabilize TAG by coating leaf lipid droplets and reducing accessibility to enzymes that might otherwise promote TAG turnover (Vanhercke *et al*., 2014; Winichayakul *et al*., 2013).

Our results show that constitutive expression of tung *FADX* in leaves of *Arabidopsis* resulted in low accumulation of ESA in phospholipids (<1% of fatty acids), revealing that leaf tissues do indeed contain robust mechanisms for exclusion of conjugated fatty acids from cellular membranes. The plant lines expressing *FADX* in leaves also displayed poor plant growth, however, indicative of possible cytotoxicity in leaves. By contrast, co‐expression of *FADX* and tung *DGAT2* resulted in a strong synergistic increase in total *Arabidopsis* leaf oil content, significantly improved channelling of ESA into TAG, and suppressed the poor growth phenotype observed with FADX alone. Furthermore, there were no observed negative effects on seed development or germination. Taken together, these results open a new avenue for producing high amounts of oil in plant leaves that is coupled with accumulation of unusual, oxidized fatty acids. The results should serve as a useful guide for production of other types of high‐value oils in the leaves of plants.

## Results

### Generation of high‐leaf‐oil plant lines expressing tung *FADX*

High‐leaf‐oil plant lines were generated by transforming wild‐type (Col‐0) (WT), *cgi‐58*,* pxa1*, or *cgi‐58*/*pxa1* double‐mutant *Arabidopsis* plants with either an empty binary plasmid or the same plasmid expressing (via constitutive promoters) tung *DGAT1*,* DGAT2*,* DGAT1*/*OLEOSIN* (*OLEO*), or *DGAT2*/*OLEO*. Seeds were selected on Basta, and then, resistant plants were advanced to the T_2_ stage to identify single copy (or closely spaced multicopy) insertions. Plants of homozygous T_3_ lines were then analysed for elevated leaf oil (TAG) content by thin‐layer chromatography (TLC), transgene expression determined by RT‐PCR, and leaf cytosolic lipid droplets visualized using confocal microscopy (Figure S1). Notably, some of the genotype/transgene combinations expressing tung *DGAT1* did not show elevated TAG content (Figure S1a), and thus, only a subset of lines were used for subsequent transformation with either a second empty binary plasmid or the same plasmid constitutively expressing tung *FADX* (Table S1). Transgenic seeds were selected on hygromycin, followed by progeny analysis and isolation of homozygous T_3_ lines (Table S1). Analysis of leaf lipids using gas chromatography with flame ionization detector (GC/FID) revealed a wide distribution in the number of plants showing at least traces of ESA in leaf lipids (Table S1), but for several genotype/transgene combinations, particularly the *cgi‐58/pxa1* double mutants and those expressing tung *DGAT1*, no lines with ESA were recovered. As such, we reduced the number of lines analysed to a subset that would allow for more direct comparisons of effects of genetic background and transgene combination on ESA production and accumulation. These included WT, *cgi‐58,* and *pxa1* mutant backgrounds transformed with either empty plasmid, *FADX* alone, *DGAT2* alone, or a combination of *FADX* and *DGAT2*. Notably, no transgenic lines were generated with *FADX* alone in the *pxa1* mutant background, and thus, this combination was not included in the analysis.

### Co‐expression of tung *FADX* and *DGAT2* results in a synergistic increase in total leaf oil content and ESA accumulation

**Figure 1:**
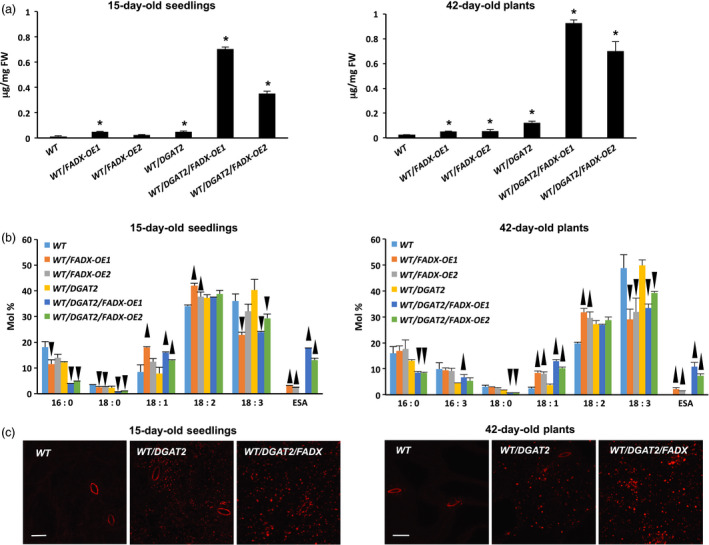
Analysis of neutral lipids and lipid droplets in *Arabidopsis* WT plant lines. (a) Content of neutral lipids in 15‐day‐old seedlings and in mature, fully expanded leaves of 42‐day‐old, soil‐grown plants (mean ± SD,* n* = 3; asterisks denote significant difference from respective empty‐vector control at *P *=* *0.05). (b) Fatty acid composition of neutral lipids in 15‐day‐old seedlings and in mature leaves of 42‐day‐old plants (mean ± SD,* n* = 3; up and down arrowheads denote values significantly higher or lower, respectively, compared to the respective empty‐vector control at *P *=* *0.05). (c) Confocal fluorescence micrographs of Nile red‐stained lipid droplets in 15‐day‐old seedlings and mature leaves of 42‐day‐old plants. Scale bar = 20 μm.

**Figure 2:**
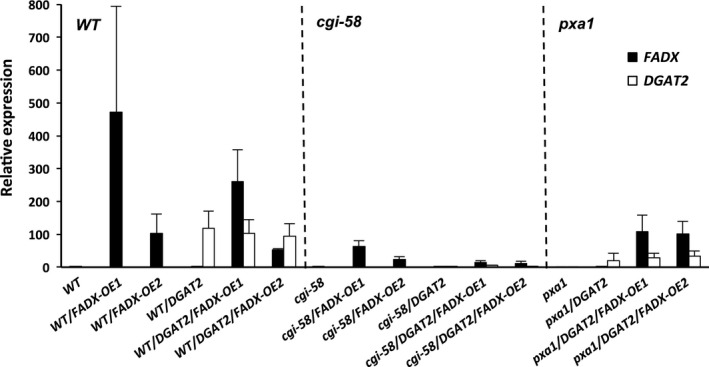
qRT‐PCR analysis of ectopically expressed tung *FADX* and *DGAT2* transcripts relative to endogenously expressed *Arabidopsis ACTIN8* in 15‐day‐old seedlings (mean ± SD,* n* = 3).

Analysis of lipids in 15‐day‐old *Arabidopsis* seedlings and in mature, fully expanded leaves of 42‐day‐old plants revealed that total neutral lipid content was moderately, yet significantly increased in WT plants expressing *FADX* or *DGAT2* alone, but co‐expression of *FADX* and *DGAT2* together resulted in a substantial, synergistic increase in neutral lipid content (Figure 1a). The increase in neutral lipids was not likely due to differences in transgene expression, as *DGAT2* expression was fairly consistent between all of the plant lines examined and *FADX* expression showed no correlation with neutral lipid content (Figure 2). Analysis of fatty acid composition (Figure 1b) further revealed that co‐expression of *FADX* and *DGAT2* also increased the percentage of ESA in neutral lipids in both 15‐day‐old seedlings and leaves of 42‐day‐old plants (Figure 1b), accounting for approximately 2% in lines expressing *FADX* alone, and 12% in lines co‐expressing *FADX* and *DGAT2* together. Notably, the percentage of oleic acid (18 : 1) was also increased in lines expressing *FADX* (Figure 1b), which is often observed in plants engineered for expression of divergent FAD2 enzymes (Broun and Somerville, 1997; Cahoon *et al*., 1999; Singh *et al*., 2001). It is generally believed that the diverged FAD2 inhibits endogenous FAD2 activity through direct protein–protein interaction and/or product‐mediated inhibition (Lou *et al*., 2014). A different mechanism might also be involved here, however, as lines with the highest percentage of 18 : 1 in leaves were reduced primarily in linolenic acid (18 : 3), rather than linoleic acid (18 : 2) (Figure 1b), the product of FAD2. The increases in total neutral lipid content of engineered lines were further supported by confocal microscopy, which revealed an increase in lipid droplet abundance in *DGAT2*‐expressing plants that was further enhanced by co‐expression with *FADX* (Figure 1c).

**Figure 3:**
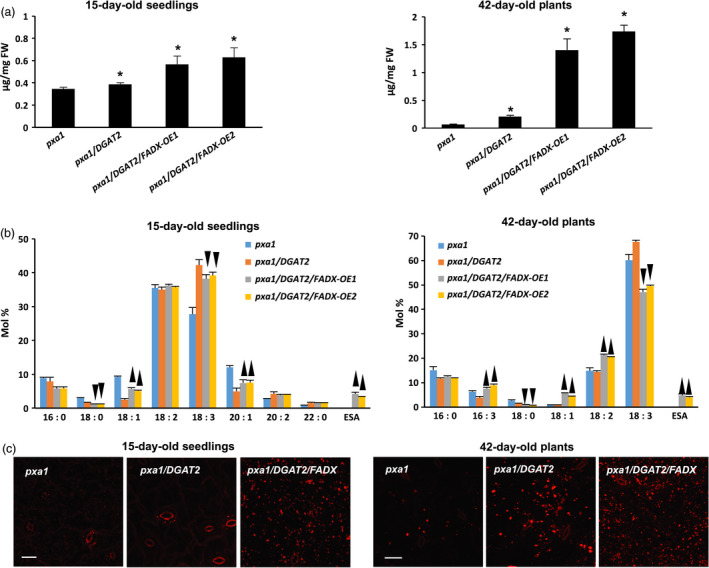
Analysis of neutral lipids and lipid droplets in *Arabidopsis pxa1* mutant plant lines. (a) Content of neutral lipids in 15‐day‐old seedlings and in mature, fully expanded leaves of 42‐day‐old, soil‐grown plants (mean ± SD,* n* = 3; asterisks denote significant difference from respective empty‐vector control at *P *=* *0.05). (b) Fatty acid composition of neutral lipids in 15‐day‐old seedlings and in mature leaves of 42‐day‐old plants (mean ± SD,* n* = 3; up and down arrowheads denote values significantly higher or lower, respectively, compared to the respective empty‐vector control at *P *=* *0.05). (c) Confocal fluorescence micrographs of Nile red‐stained lipid droplets in 15‐day‐old seedlings and in mature leaves of 42‐day‐old plants. Scale bar = 20 μm.

Similar increases in neutral lipid and ESA contents were observed when *FADX* and *DGAT2* were co‐expressed in *pxa1* and *cgi‐58* mutant backgrounds (Figure 3 and S2). Results with *cgi‐58* lines were not, however, as pronounced as in WT or *pxa1* transgenics (Figure S2), likely due to the relatively low level of transgene expression (Figure 2). The neutral lipid content of 15‐day‐old *pxa1* seedlings was already elevated in comparison with 15‐day‐old WT plants (compare Figure 3a and 1a), which is consistent with an inability of *pxa1* mutant plants to degrade fatty acids (Kunz *et al*., 2009; Slocombe *et al*., 2009; Zolman *et al*., 2001). Analysis of fatty acid composition further revealed the presence of very‐long‐chain fatty acids (e.g. 20 : 1, 20 : 2, and 22 : 0) in neutral lipids of 15‐day‐old seedlings (Figure 3b), likely due to the persistence of seed storage oils in these tissues. Co‐expression of *FADX* and *DGAT2*, however, led to an increase in neutral lipid content above and beyond that observed in either *pxa1* seedlings or those expressing *DGAT2* (Figure 3a). Notably, the total neutral lipid content of *pxa1*/*DGAT2*/*FADX* 15‐day‐old seedlings was similar to that of WT/*DGAT2*/*FADX* seedlings (i.e. ~0.6 μg/mg FW). In mature, fully expanded leaves of 42‐day‐old plants, however, the neutral lipid content of *pxa1*/*DGAT2*/*FADX* plants was nearly doubled that of WT/*DGAT2*/*FADX* plants (i.e. ~1.5 μg/mg FW and ~0.7 μg/mg FW, respectively) (compare Figures 3a and 1a), indicating that loss of PXA1 function leads to a further increase in steady‐state accumulation of neutral lipids as the plants age. Like WT transgenic lines, analysis of fatty acid composition showed an increase in percentage of 18 : 1 in *pxa1* lines expressing *FADX* and *DGAT2* (Figure 3b), and confocal microscopy revealed comparable increases in lipid droplet abundance (Figure 3c).

### Engineering ESA production in leaves has no effect on total polar lipid content, but causes changes in fatty acid composition

**Figure 4:**
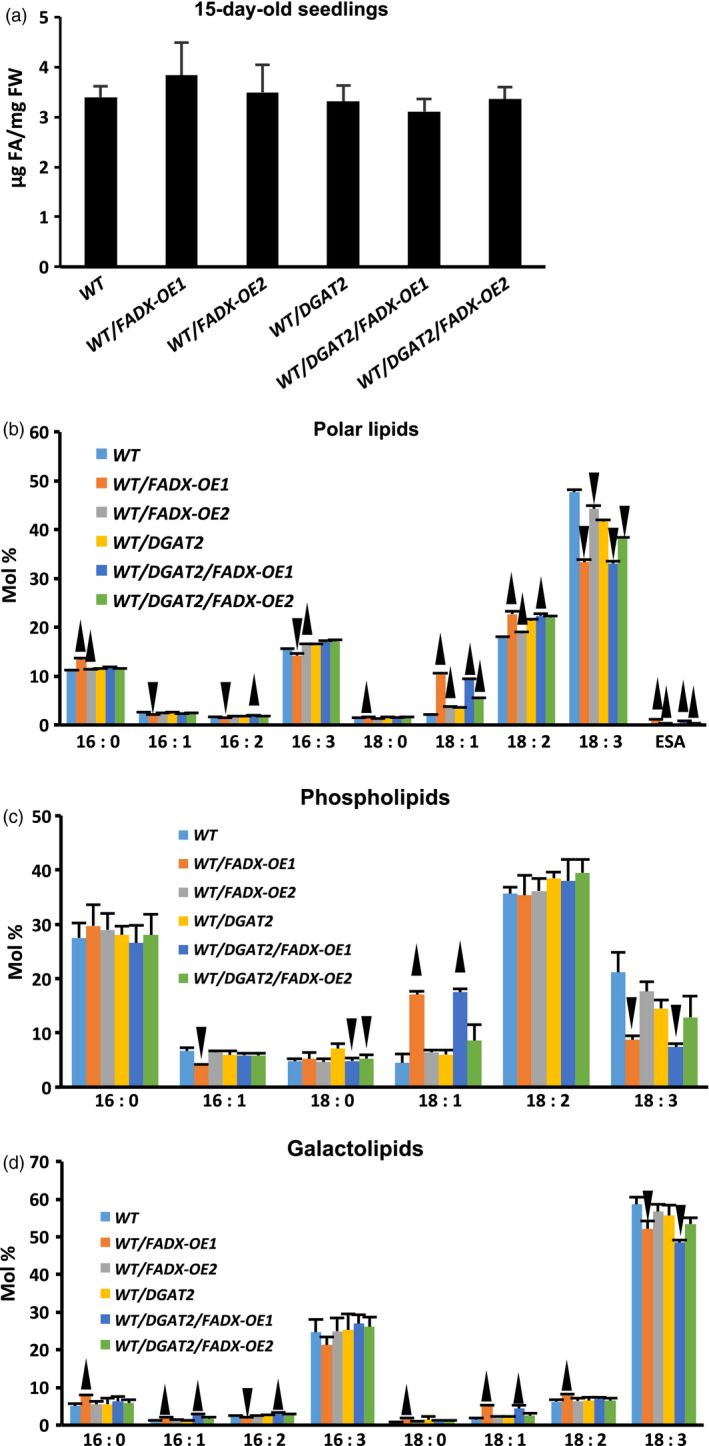
Analysis of polar lipids in 15‐day‐old seedlings of *Arabidopsis* WT plant lines. Total polar lipid content (a) and fatty acid composition of total polar lipids (b), phospholipids (c), and galactolipids (d), in 15‐day‐old seedlings of WT plant lines (mean ± SD,* n* = 3; up and down arrowheads denote values significantly higher or lower, respectively, compared to the respective empty‐vector control at *P *=* *0.05).

We next investigated whether there were any changes in polar lipid content and composition of leaves engineered for production of ESA. As shown in Figure 4a for 15‐day‐old seedlings, and Figure S3a for mature, fully expanded leaves of 42‐day‐old plants, there were no significant changes in total polar lipid content in any of the WT plant lines investigated. Inspection of fatty acid composition of total polar lipids, however, revealed trace amounts of ESA (<1% of total fatty acids) in WT lines expressing *FADX*, with or without co‐expression of *DGAT2* (Figures 4b and S3b). Notably, the majority of WT lines expressing *FADX*, with or without *DGAT2*, also showed elevated 18 : 1 content and reduced 18 : 3 content, which is consistent with results observed for neutral lipids (Figure 1b). Similar results were observed for analysis of polar lipids in *pxa1* and *cgi‐58* mutant plant lines (Figures S4 and S5, respectively).

To determine whether the changes in fatty acid composition were present primarily in phospholipids or galactolipids, the total polar lipid fraction from 15‐day‐old WT transgenic seedlings was separated by TLC then major lipid classes were isolated and analysed by GC‐FID. As shown in Figure 4, the high oleate (18 : 1) and reduced linolenate (18 : 3) phenotype was more pronounced in phospholipids (Figure 4c) than galactolipids (Figure 4d). Similar results were observed for phospholipids and galactolipids of *pxa1* mutant plant lines (Figure S4). These data are consistent with the known localization of FADX in the ER (Dyer *et al*., 2002) and activity of conjugases towards PC‐linked substrates (Liu *et al*., 1997); the changes in galactolipid composition likely reflect the extensive exchanges of glycerolipids known to occur between ER and chloroplast membranes (Benning *et al*., 2006; Browse *et al*., 1986). The consistency of high 18 : 1 and reduced 18 : 3 in both phospholipid and neutral lipid fractions of engineered lines further supports a metabolic relationship between these two lipid classes for production of neutral lipids containing ESA.

### Production of ESA in plant leaves is detrimental to plant growth, but is improved by co‐expression of *FADX* and *DGAT2*

**Figure 5:**
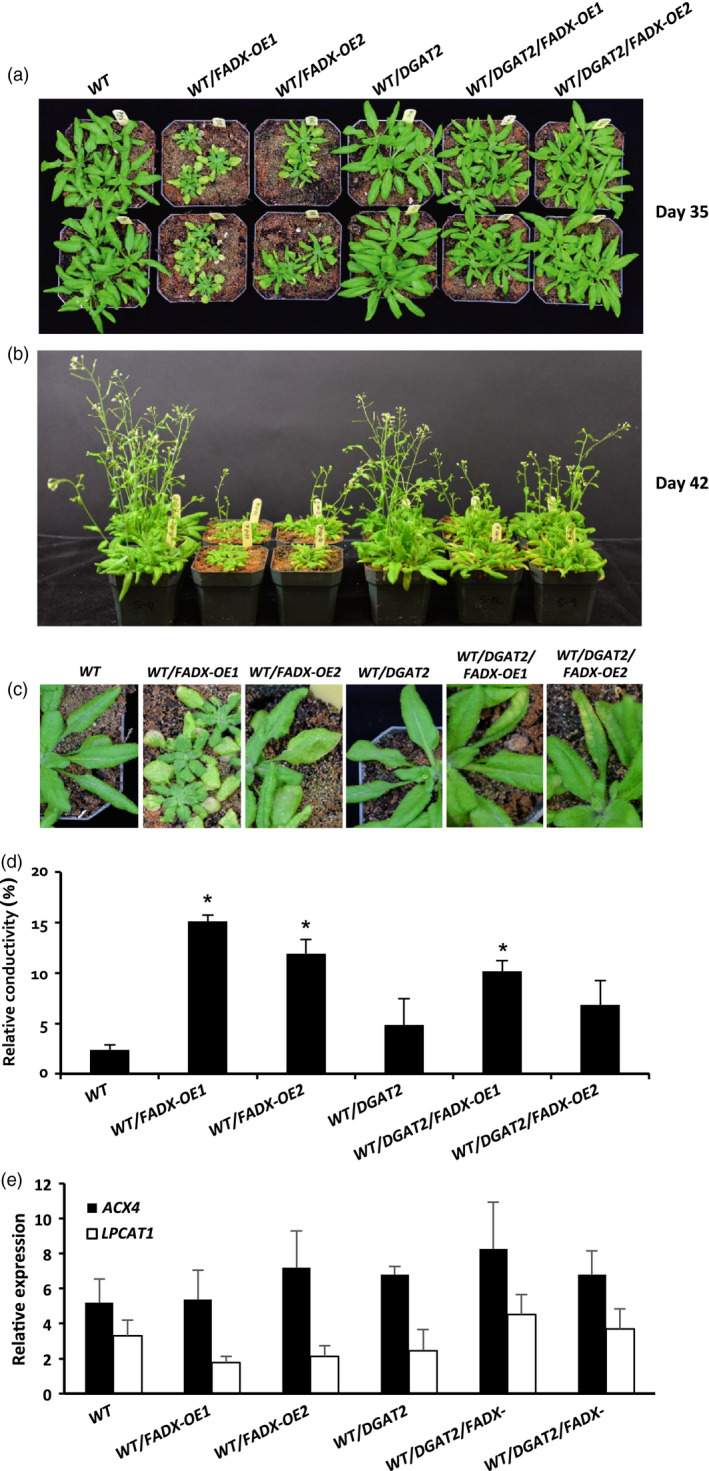
Phenotypes of *Arabidopsis* WT plant lines expressing tung *FADX* and/or *DGAT2*. Images of 35‐day‐old (a) and 42‐day‐old (b) soil‐grown plants, and mature, fully expanded leaves of 35‐day‐old plants (c). Electrolyte leakage assay (d) of mature leaves from 35‐day‐old plants (mean ± SD,* n* = 3; asterisks denote significant difference from respective empty‐vector control at *P *=* *0.05). (e) qRT‐PCR analysis of *Arabidopsis LPCAT1* and *ACX4* transcripts in leaves of 43‐day‐old plants relative to *Arabidopsis ACTIN8* (mean ± SD,* n* = 3).

WT plants expressing *FADX* alone often exhibited reduced plant growth and yellowing of leaves in comparison with empty plasmid controls, with obvious differences apparent by 35 days after germination (Figure 5a–c). Co‐expression of *FADX* and *DGAT2* in the WT background, however, suppressed the phenotype, resulting in more normal‐sized plants and less leaf discoloration (Figure 5a–c). Further, measurement of ion leakage, an indicator of cell death (Kawai‐Yamada *et al*., 2004), showed higher relative conductivity in leaves expressing *FADX* alone, and the phenotype was partially suppressed by co‐expressing *DGAT2* (Figure 5d). Measurement of endogenous *ACYL‐CoA OXIDASE 4* (*ACX4)* and *LYSOPHOSPHATIDYLCHOLINE ACYLTRANSFERASE 1* (*LPCAT1*) expression, which is induced during plant senescence (Troncoso‐Ponce *et al*., 2013), showed no obvious changes in any of the lines examined (Figure 5e). These data are generally consistent with a model whereby ESA is synthesized in the phospholipids of the ER, and then removed from membranes as a free fatty acid or acyl‐CoA, which promotes cytotoxicity, and DGAT2 reduces cytotoxic effects by more effectively capturing ESA and sequestering it in TAG.

**Figure 6:**
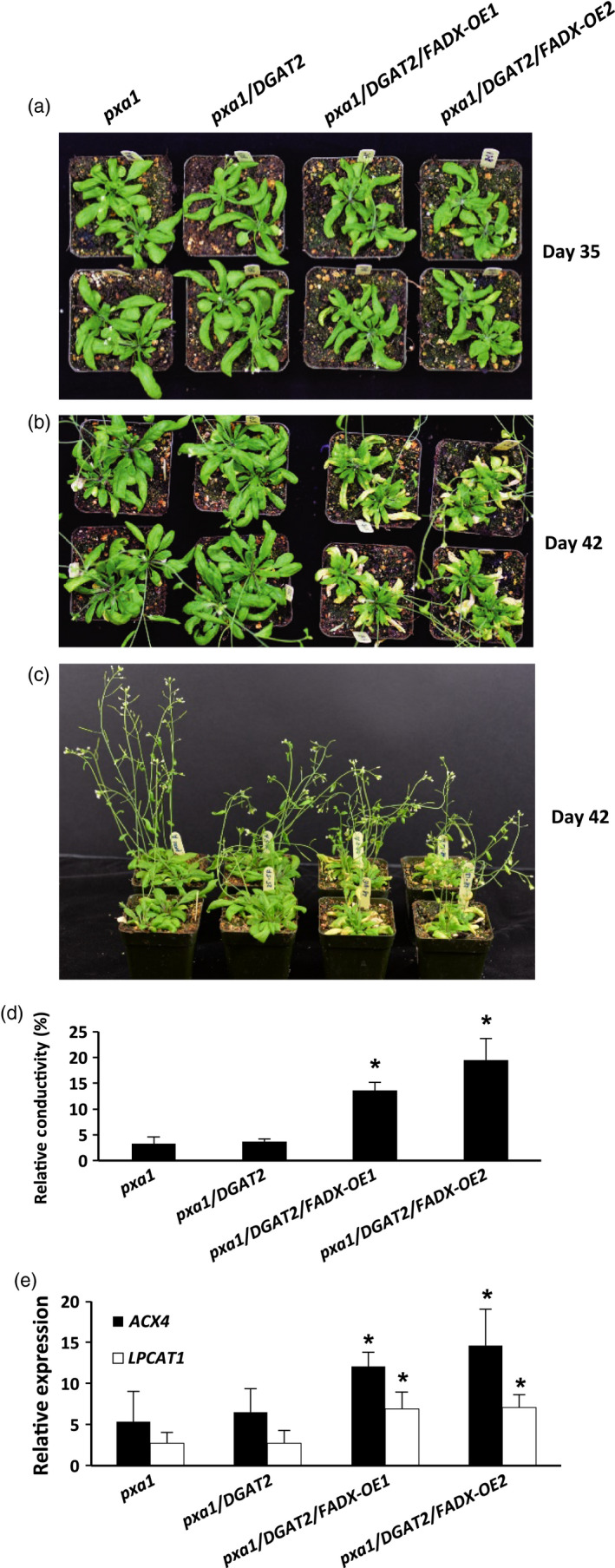
Phenotypes of *Arabidopsis pxa1* mutant plant lines expressing tung *FADX* and/or *DGAT2*. Images of 35‐day‐old (a) and 42‐day‐old (b and c) soil‐grown plants. Electrolyte leakage assay (d) of mature, fully expanded leaves from 37‐day‐old plants. (e) qRT‐PCR analysis of *Arabidopsis LPCAT1* and *ACX4* transcripts in mature leaves of 43‐day‐old plants relative to *Arabidopsis ACTIN8*. Values in (d) and (e) represent mean ± SD,* n* = 3; asterisk denotes significant difference from respective empty‐vector control at *P *=* *0.05.

Consistent with this premise, the cytotoxic effects were more pronounced in *pxa1* mutant plant lines (Figure 6). Plants harbouring mutations in *PXA1* are known to be more sensitive to the cytotoxic effects of free fatty acids, due in large part to their inability to degrade fatty acids (Kunz *et al*., 2009). In *pxa1* lines expressing *FADX* and *DGAT2* together, plant size was similar to *DGAT2*‐only controls (Figure 6a and b), but the leaves of *pxa1*/*DGAT2*/*FADX* plants showed more obvious yellowing and necrosis in comparison with WT/*DGAT2*/*FADX* lines (compare Figures 6 and 5). Furthermore, ion leakage assays revealed that relative conductivity remained high in *pxa1*/*DGAT2*/*FADX* lines (Figure 6d), and *ACX4* and *LPCAT1* were induced in comparison with controls (Figure 6e). Taken together, these data indicate that *pxa1* mutants are more sensitive than WT to ESA production and accumulation. By contrast, *cgi‐58* transgenic plants showed essentially normal plant growth and development (Figure S6), again likely due to the relatively low level of *FADX* and *DGAT2* expression in this mutant background (Figure 2).

### Engineering the production of ESA in plant leaves has essentially no effect on seeds

**Figure 7:**
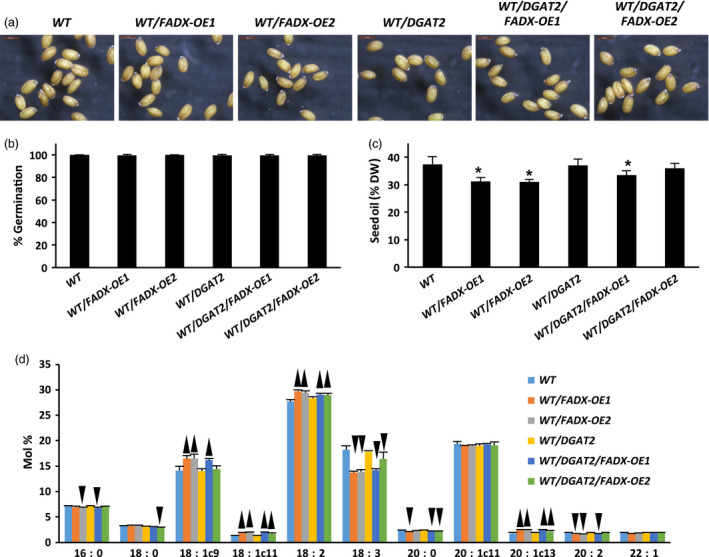
Properties of seeds derived from *Arabidopsis* WT plant lines expressing tung *FADX* and/or *DGAT2*. (a) Images of mature, dry seeds (at 3.5× magnification). (b) Percentage of seed germination. (c) Seed oil content determined by NMR (mean ± SD,* n* = 5; asterisks denote values significantly different from respective empty‐vector control at *P *=* *0.05). (d) Fatty acid composition of seed oil (mean ± SD,* n* = 5; up and down arrowheads denote values significantly higher or lower, respectively, compared to the respective empty‐vector control at *P *=* *0.05).

One of the major challenges for producing high amounts of ESA in transgenic seeds is that the seeds are often shrunken and wrinkled, and have poor germination rates (Cahoon *et al*., 1999, 2006). To determine whether the engineering of ESA production in leaves had any effects on seeds, we examined seed morphology, germination potential, and oil content and composition. As shown in Figure 7a, seeds of WT transgenic lines showed similar overall morphology, size and pigmentation in comparison with the empty plasmid control seeds. The engineered seeds also showed normal germination rates, which was nearly 100% germination frequency for all plant lines (Figure 7b). Examination of total oil content revealed that some of the plant lines had reduced seed oil (Figure 7c), which might be due to the smaller leaf sizes and/or necrosis and senescence in the associated transgenic plants (Figure 5), but no ESA, or only trace amounts, was detected in seed lipids (Figure 7d). Similar results were observed for seeds derived from both *pxa1* and *cgi‐58* mutant plant lines (Figure S7 and S8, respectively).

## Discussion

Production of industrially important fatty acids in engineered seeds is often limited by their inefficient exclusion from phospholipid membranes, resulting in reduced flux of the fatty acid to storage oil and, particularly in the case of conjugated fatty acids, negative effects on seed development and germination (Cahoon *et al*., 2006; Napier *et al*., 2014). Here, we examined the possibility of coupling the production of unusual fatty acids in plant leaves with accumulation of high amounts of TAG, thereby developing a novel strategy for producing high‐value oils in plants. The engineering approach was designed, in part, based on prior studies that examined constitutive expression of fatty acid hydroxylases and conjugases in transgenic plants, which showed efficient exclusion of unusual fatty acids from phospholipids of leaves (Iwabuchi *et al*., 2003; van de Loo *et al*., 1995). Expression of tung *FADX* in WT leaves resulted in low accumulation of ESA in polar lipids (<1% weight of total fatty acids; Figure 4), consistent with these prior observations. While it is possible that the low amounts of ESA in leaf lipids are due to low FADX activity, the negative effects of *FADX* expression on plant growth and development (Figure 5a–c), coupled with the observation that co‐expression with *DGAT2* significantly enhances TAG and ESA accumulation (Figure 1a and b), would more likely suggest that ESA is synthesized in phospholipids, as expected, and then rapidly excluded from membranes, possibly by phospholipase A2 (PLA2) or the reverse reaction catalysed by LPCAT (Bates, 2016). Indeed, LPCATs are known to show preferential activity towards oxidized fatty acids in PC (Lager *et al*., 2013), which might suggest a role in exclusion of ‘unusual’ fatty acids that are otherwise disruptive to cellular membranes. This activity is likely to be particularly important in photosynthetic tissues, where oxidative stress often contributes to lipid peroxidation (Triantaphylidès *et al*., 2008). Regardless, the identification of the enzyme(s) responsible for exclusion of ESA from phospholipids in leaves would be a useful tool for increasing flux of ESA in engineered plants.

The low accumulation of ESA in neutral lipids of plant leaves expressing tung *FADX* alone (Figure 1b) suggests also that the endogenous acyltransferases catalysing TAG synthesis in leaves do not effectively metabolize substrates containing ESA. Both phospholipid:diacylglycerol acyltransferase1 (PDAT1) and DGAT1 are known to play a role in production of TAG in leaves (Zhang *et al*., 2009), and studies employing *tgd1* mutants of *Arabidopsis* (Fan *et al*., 2013, 2014) have shown that PDAT1 is required for synthesis of TAGs that serve as a buffer against excess lipids and cytotoxic free fatty acids. Given that only trace amounts of ESA were observed in the neutral lipids of leaves expressing *FADX* alone (Figure 1b), it is unlikely that ESA was excluded from phospholipids by the activity of endogenous PDAT. Rather, ESA is probably removed first by a PLA2 and/or the reverse reaction of LPCAT, and then inefficiently used by endogenous DGAT for formation of TAG. Notably, the stunted size and appearance of white, necrotic spots and leaf yellowing in lines expressing tung *FADX* alone (Figure 5c) is similar to the phenotype of *tgd1‐1*/*pdat1‐2* mutant plants (Fan *et al*., 2013), which also contained elevated amounts of diacylglycerol (DAG) and free fatty acids. While we did not measure ESA content in the free fatty acid fraction, due to the very labile nature of ESA as a free acid (Yang *et al*., 2009), the relative increase in cytotoxic effects of *FADX* when expressed in the *pxa1* mutant background (Figure 6) would further support a model involving fatty acid cytotoxicity (Kunz *et al*., 2009).

### Co‐expression of tung *FADX* and *DGAT2* improves plant growth, increases leaf oil content, and greatly enhances channelling of ESA into neutral lipids

**Figure 8:**
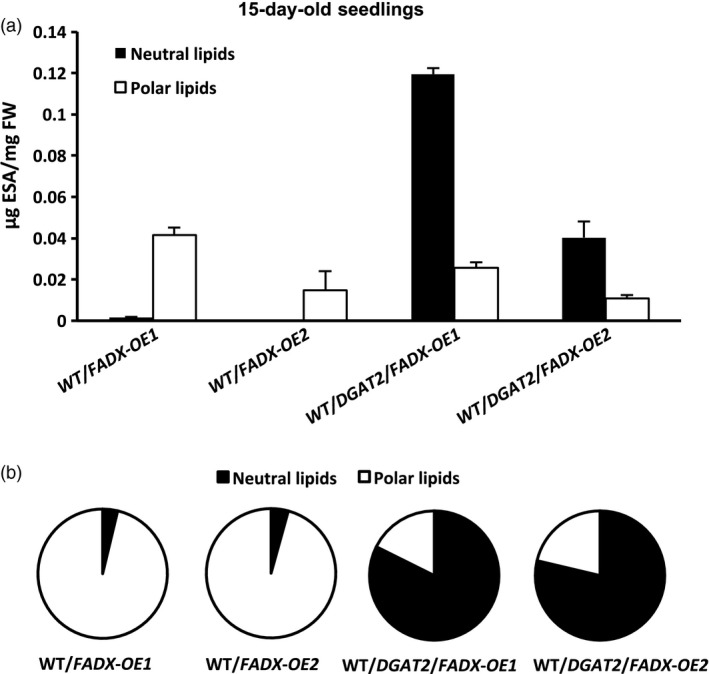
Distribution of ESA in lipids of 15‐day‐old *Arabidopsis* WT plant lines. (a) Content of ESA in neutral and polar lipids, plotted based on total mass amounts (mean ± SD,* n* = 3). (b) Percentage of ESA in neutral and polar lipids, plotted by setting the total mass amounts of ESA for each plant line in (a) to 100%.

Expression of tung *DGAT2* alone in plant leaves resulted in at least a doubling in neutral lipid content (Figure 1a), consistent with prior studies that employed DGATs from other source species for increasing oil content in leaves (Andrianov *et al*., 2010; Bouvier‐Navé *et al*., 2000; Vanhercke *et al*., 2014). Co‐expression of tung *FADX* and *DGAT2*, however, resulted in a synergistic increase in neutral lipid content, increase in ESA accumulation, and improved plant growth and development (Figures 1 and 5). These results support a model whereby ESA is first excluded from phospholipids, and then effectively captured and channelled into TAG by tung DGAT2. By plotting the amount of ESA in lines expressing *FADX* alone versus *FADX* and *DGAT2*, as a percentage of total ESA present in polar or neutral lipids (Figures 8 and S9), it becomes readily apparent that tung DGAT2 helps to partition ESA from polar lipids into the neutral lipid fraction. This partitioning likely contributes to improved plant growth and development through sequestration of ESA metabolites that would otherwise be toxic.

### Blocking fatty acid degradation by disrupting PXA1 elevates steady‐state accumulation of neutral lipids and ESA, but also has undesirable effects on plant growth and development

Prior research indicated that unusual fatty acids are often degraded in engineered seeds, resulting in a futile cycle of synthesis and turnover that limits their steady‐state accumulation (Eccleston and Ohlrogge, 1998). Therefore, we expected that disruption of fatty acid breakdown would be an important strategy for elevating unusual fatty acid content in leaves. Somewhat surprisingly, this was not entirely the case. Comparisons of 15‐day‐old WT and *pxa1* lines co‐expressing tung *FADX* and *DGAT2* revealed similar increases in total neutral lipid and ESA content (Figures 1a and 3a). Comparison of lipids in 42‐day‐old plants, however, revealed additional increases in total neutral lipid content in *pxa1* transgenic lines relative to WT lines, although trends in polar lipids remained the same (Figures 1a and 3a; Figures 4 and S4). These data indicate that *pxa1* mutants can indeed be used to increase neutral lipid content above and beyond that observed in WT lines, but the plants are significantly less healthy at this stage of development (compare Figures 5 and 6). Furthermore, in addition to a role in fatty acid breakdown, PXA1 is known to function in the transport of lipid hormone precursors into peroxisomes for their subsequent β‐oxidation to produce indole‐3‐acetic acid and jasmonic acid, and thus, *pxa1* mutants are compromised in some aspects of plant growth, development, and stress response (Dave *et al*., 2011; Theodoulou *et al*., 2005; Zolman *et al*., 2001). And finally, *pxa1* mutant plants are also unable to degrade seed storage oil and thus require an exogenous source of sugar for seedling establishment (Footitt *et al*., 2002; Hayashi *et al*., 2002). As such, any engineering strategy that employs disruption of PXA1 must account for the difficulties with seed germination and establishment. One potential mechanism to circumvent these difficulties is to use inducible RNAi methods to silence *PXA1* expression at particular stages of development, and/or only in certain tissues (Kim *et al*., 2015).

### Future directions

To further increase oil and ESA contents in leaves, obvious next steps include an increase in fatty acids available for TAG synthesis, which can be accomplished using various ‘push’‐related strategies, including ectopic expression of the transcription factor WRINKLED1, which activates multiple genes involved in fatty acid synthesis (Vanhercke *et al*., 2013a), suppression of ADP‐glucose pyrophosphorylase, which alters flux of carbon from starch into fatty acid production (Sanjaya *et al*., 2011), or over‐expression of FAX1 (fatty acid export 1), which enhances transport of fatty acids out of chloroplasts (Li *et al*., 2015). Other enzymes for increased channelling of ESA into TAG would also likely include tung homologs of glycerol‐3‐phosphate acyltransferase 9 (Shockey *et al*., 2016; Singer *et al*., 2016) and lysophosphatidyl acyltransferase 2 (Chen *et al*., 2016). Additional enzymes, such as phosphatidylcholine diacylglycerol cholinephosphotransferase, might also be required to increase flux of ESA‐containing metabolites through PC (Bates and Browse, 2011; Bates *et al*., 2012; Hu *et al*., 2012).

While the production of elevated oil in plant leaves effectively increases the caloric content of biomass crops, which is desirable for biofuel or animal feed production (Horn and Benning, 2016), there is also potential for altering plant/pest interactions. For instance, feeding studies have demonstrated increased caterpillar weights when insects were fed a diet of leaves containing elevated oil content (Sanjaya *et al*., 2013), and thus, strategies for mitigating pest predation should be considered. Unusual fatty acids, including hydroxy or acetylenic, have potential antiphysiological effects (Cahoon *et al*., 2003; Tunaru *et al*., 2012). As such, the production of unusual fatty acids in leaf oil might serve a dual purpose in elevating oil content in leaves, while at the same time discouraging plant–pest interactions.

Overall, the demonstration provided here, showing a synergistic relationship between an enzyme for unusual fatty acid synthesis (FADX) and an enzyme for selective channelling into TAG (DGAT2), should serve as a useful guide for production of other industrially important fatty acids in plants. There are many other divergent FAD2 enzymes responsible for synthesis of a wide array of fatty acid structures, including epoxy, hydroxy, and acetylenic fatty acids (Lee *et al*., 1998; van de Loo *et al*., 1995; Shanklin and Cahoon, 1998), and DGAT2 enzymes are known to be important for their accumulation in engineered seeds (Burgal *et al*., 2008; van Erp *et al*., 2011; Li *et al*., 2010a). These observations suggest that other, structurally diverse fatty acids can be produced in plant leaves by matching the fatty acid‐modifying enzyme with the acyl‐CoA‐dependent DGAT from the same source plant species.

## Experimental procedures

### Gene cloning and construction of tung gene binary plasmids

The contents and available restriction sites for all cloning and binary plasmids described below are described in Shockey *et al*. (2015), except where noted. The full‐length open reading frames (ORFs) for myc‐epitope‐tagged tung DGAT1 and DGAT2 were initially cloned into the dual CaMV 35S promoter/terminator shuttle plasmid K34. The two promoter:tung *DGAT1/2*:terminator *Asc*I cassettes were then ligated separately into the *Asc*I site of the binary plasmid B9. The ORF for tung *OLEOSINII,* which was identified in the tung seed cDNA 454 sequencing project (Pastor *et al*., 2013), was cloned into the nos promoter‐nos terminator cassette in cloning plasmid K33. Thereafter, the *Asc*I promoter:*OLEOSINII*:terminator cassette from this plasmid was ligated into the *Mlu*I site of each of the tung *DGAT*‐B9 binary plasmids. In this study, B9 is also referred to as EV1 (empty vector 1) because it served as the negative control plasmid used in the first round of plant transformations. The haemagglutinin (HA)‐epitope‐tagged tung *FADX* ORF was cloned into the binary plasmid pMDC32 (Curtis and Grossniklaus, 2003) using the *Asc*I and *Sac*I sites located between the dual CaMV 35S promoter and terminator. Empty vector 2 (EV2), which was used in the second round of plant transformations, was constructed by removal of the attR1‐ccdB‐attR2 cassette from pMDC32 as an *Asc*I‐*Sac*I fragment, followed by restoration of blunt ends by Klenow fill‐in, and self‐ligation.

### Plant material, growth conditions, transformation, and seed germination

*Arabidopsis* lines used in this study were WT Columbia‐0 ecotype and derivatives thereof, including the T‐DNA insertional mutant line, *cgi‐58* [SALK_136871] (James *et al*., 2010), *pxa1*, an ethyl methanesulfonate‐generated, splice variant mutant (Zolman *et al*., 2001), and *cgi‐58/pxa1*, a double mutant generated by crossing *pxa1* and *cgi‐58* plants (Park *et al*., 2013). Plants were grown on soil in a growth chamber set for 16‐h light/8‐h dark cycle at 22 °C, 40% RH, and 50 μE/m^2^/s. Seeds were surface‐sterilized and plated on ½ MS media (Murashige and Skoog, 1962) solidified with 0.8% Gelzan (Sigma‐Aldrich) with (for *pxa1* mutants) or without 1% sucrose, and antibiotics, as specified. After 3 days of stratification in the dark at 4 °C, plates with seeds were moved to the growth chamber with growth conditions as described above. Seedlings were transferred to soil at 10 days after stratification or harvested for analysis at 15 days after stratification.

*Agrobacterium*‐mediated transformation of *Arabidopsis* plants was performed using the floral dip method of Clough and Bent (1998), using *A. tumefaciens* strain GV3101, as described previously (Cai *et al*., 2015). Bulk T_1_ seeds were collected from mature transformed T_0_ plants and sown on soil wetted with 0.15% final concentration Basta (ChemService Inc.). At least 24 individual T_1_ plants were chosen at random from each *Agrobacterium*/plant genotype transformation and transplanted to individual pots and grown to maturity. Segregating T_2_ seed samples were used to further select homozygous T_3_ lines for further analysis. High‐performing lines (as evidenced by TLC, qRT‐PCR, and confocal microscopy) were re‐transformed with *Agrobacterium* strains carrying either hygromycin‐resistant binary plasmid pMDC32 with or without the HA‐tagged tung *FADX* gene. Additional rounds of selection for hygromycin‐resistant double transgenic lines were carried out as described in Harrison *et al*. (2006).

Seed germination was evaluated by first sterilizing, plating, and stratifying seeds as described above, and then, plates were moved to a growth chamber with the same conditions as before. The assay was performed in triplicate, and germination was scored by radical emergence after 3 days.

### Lipid analysis

For semi‐quantitative analysis of neutral lipids from vegetative tissues by TLC, total lipids were extracted from 50 mg FW of mature (fully expanded, but not senescing) leaves of soil‐grown 42‐day‐old plants using a hexane/isopropanol method (Hara and Radin, 1978; Gidda *et al*., 2016). Total lipids in chloroform were separated on a silica TLC plate (Merck) using hexane: diethyl ether: acetic acid (70:30:1, v/v/v), stained with 0.05% primuline in 80% acetone and visualized under UV light. C17:0 TAG (Sigma‐Aldrich) was used as an external standard.

For analysis of content and fatty acid composition of neutral and polar lipids from vegetative tissues, total lipids were extracted from 500 mg FW of 15‐day‐old seedlings grown on ½ MS medium (and 1% sucrose for *pxa1* mutants) or from 500 mg of mature, fully expanded leaves from 42‐day‐old soil‐grown plants, with addition of C17:0 TAG (Sigma‐Aldrich) and C15:0 PC (Avanti Polar Lipid, Inc.) as internal standards. Total lipid extracts in hexane were separated into neutral and polar lipids on solid‐phase extraction cartridges (Supelco Discovery DSC‐Si 6 mL), as described (Gidda *et al*., 2016). To prepare fatty acid methyl esters (FAMEs), 0.5 mL of 0.5 N sodium methoxide solution in methanol was added to neutral or polar lipid extracts, and samples were incubated at room temperature in the dark for 25 min. The reaction was quenched with 1 mL of saturated NaCl solution in water, and FAMEs were extracted with 1 mL of hexane. FAME samples were analysed on an Agilent HP 6890 series GC system equipped with a 7683 series injector and autosampler and a BPX70 (SGE Analytical Science) capillary column (10 m × 0.1 mm × 0.2 μm) with a constant pressure of 25 PSI, as described in Gidda *et al*. (2016). Compounds were identified by comparing with the GLC‐10 FAME standard mix (Sigma‐Aldrich) and FAMEs prepared from tung oil. Two‐tailed Student's *t*‐tests were used for comparisons to the respective empty‐vector control; *P *=* *0.05.

For analysis of the fatty acid composition of galactolipids and phospholipids, a portion of the polar lipid fraction from 15‐day‐old seedlings was applied on a silica TLC plate and developed in a solvent system of acetone:toluene:water (91: 30: 7.5, v/v/v), as described (Wang and Benning, 2011). Lipids were stained with 0.05% primuline in 80% acetone and visualized under UV light. Silica spots corresponding to position of galactolipids (MGDG and DGDG) and phospholipids (PC, PE, PI) were scraped and used for direct transmethylation with 1.25 m HCl in methanol at 85°C for 2 h. FAMEs were extracted with 1 mL of hexane and analysed by GC as described above.

Seed oil content was determined on 50 mg samples of dry seeds using an mq20 NMR Analyzer (Bruker). For analysis of fatty acid composition of seed oil, samples of 50 seeds were homogenized in 0.5 mL hexane on GenoGrinder (SPEX SamplePrep) for 3 min, 0.5 mL of 0.5N sodium methoxide solution in methanol was added to the homogenate, and then, samples were incubated at room temperature in the dark under the nitrogen for 25 min. Extraction and analysis of FAMEs was performed as described above.

### qRT‐PCR

For analysis of expression levels of tung transgenes, total RNA was extracted from ~100 mg of the 15‐day‐old *Arabidopsis* seedlings using the RNeasy Plant Mini Kit (Qiagen) and treated with DNase (Qiagen). Complementary DNA (cDNA) was synthesized from 1 μg of total RNA using iScript Reverse Transcription Supermix for RT‐qPCR (Bio‐RAD) following the manufacturer's protocol. Quantitative PCR of tung *DGAT1*,* DGAT2*,* OLEOSINII,* and *FADX*, and *Arabidopsis ACTIN8* or *18S* (as reference transcripts) was performed using iTaq Universal SYBR Green Supermix (Bio‐RAD) using gene‐specific forward and reverse primers (Table S2) on Bio‐RAD CFX96 Real‐Time System with C1000 Thermal cycler. All samples were also subjected to a melt curve analysis between 65 and 95°C with 0.5°C increment. For analysis of the transcript levels of *Arabidopsis ACX4*,* LPCAT1,* and *ACTIN8*, the same protocol was used except that cDNA was prepared from leaves of 43‐day‐old plants. Data were quantified using the Delta CT method, and two‐tailed Student's *t*‐tests were used for comparisons to the empty‐vector control (*P *=* *0.05).

### Microscopy

WT and transgenic 15‐day‐old *Arabidopsis* seedlings, or mature, fully expanded leaves of 28‐ or 42‐day‐old plants, were collected from the growth chamber at the end of the night cycle, when the abundance of LDs is at its highest (Gidda *et al*., 2016), and then processed for confocal microscopy, including staining with BODIPY 493/503 (Invitrogen) or Nile red (Sigma‐Aldrich) as described previously (Cai *et al*., 2015; Park *et al*., 2013). Microscopic images were acquired using a Leica DM RBE microscope with a Leica 63× Plan Apochromat oil‐immersion objective, a Leica TCS SP2 scanning head, and the Leica TCS NT software package. Nile red was excited using a 543‐nm laser and BODIPY and chlorophyll autofluorescence were excited with a 488‐nm laser. All fluorophore emissions were collected sequentially as 15 μm *Z*‐series of the adaxial surface of true leaves, and all images of cells shown are representative of at least three separate experiments.

### Electrolyte leakage assay

Relative conductivity was measured essentially as described (Wan *et al*., 2014). Briefly, three detached leaves from 35‐day‐old *Arabidopsis* plants were rinsed with deionized water and immersed in 5 mL of deionized water. Samples were gently agitated (120 rpm) for 16 h. Conductivity was measured using an Accumet Excel XL50 conductivity meter, and then, leaf samples were incubated at 95 °C for 15 min, allowed to cool to room temperature for 1 h, and the second conductivity measurement was taken. Relative conductivity was calculated as a percentage of the final conductivity measurement: RC = (Cond_16 h_/Cond_95C_) × 100.

## Supporting information


**Figure S1** Analysis of selected high‐leaf‐oil *Arabidopsis* transgenic lines. (a) TLC and gene expression analysis, with genotype/transgene combinations shown at the bottom of each bar graph. TLC analysis of lipids derived from mature, fully expanded leaves of 42‐day‐old plants are shown above the respective bar graphs showing qRT‐PCR analysis of tung *DGAT1* or *DGAT2* transcripts in 15‐day‐old seedlings relative to endogenous *Arabidopsis 18S* rRNA. Only the lanes for the top two TAG‐containing lines for each genotype/transgene combination are shown. The position of the TAG standard is shown to the left. EV1–Empty Vector 1. Asterisks denote plant lines selected for subsequent transformation with tung *FADX* or with EV2–empty vector 2. (b) Confocal fluorescence micrographs of mature, fully expanded leaves from 28‐day‐old *Arabidopsis* plant lines (as indicated by labels in panels) stained with BODIPY (lipid droplets appear green); chloroplast autofluorescence is coloured blue. Scale bar = 20 μm.

**Figure S2** Analysis of neutral lipids and lipid droplets in *Arabidopsis cgi‐58* mutant plant lines. (a) Content of neutral lipids in 15‐day‐old seedlings and in mature, fully expanded leaves of 42‐day‐old, soil‐grown plants (mean ± SD, *n* = 3; asterisks denote significant difference from respective empty‐vector control at *P *=* *0.05). (b) Fatty acid composition of neutral lipids in 15‐day‐old seedlings and in mature leaves of 42‐day‐old plants (mean ± SD, *n* = 3; up and down arrowheads denote values significantly higher or lower, respectively, compared to the respective empty‐vector control at *P *=* *0.05). (c) Confocal fluorescence micrographs of Nile red‐stained lipid droplets in 15‐day‐old seedlings of *cgi‐58* lines. Scale bar = 20 μm.

**Figure S3** Analysis of polar lipids in *Arabidopsis* WT plant lines. Content (a) and fatty acid composition (b) of polar lipids derived from mature, fully expanded leaves of 42‐day‐old, soil‐grown plants (mean ± SD, *n* = 3; up and down arrowheads denote values significantly higher or lower, respectively, compared to the respective empty‐vector control at *P *=* *0.05).

**Figure S4** Analysis of polar lipids in *Arabidopsis pxa1* mutant plant lines. Content (a) and fatty acid composition (b) of polar lipids in 15‐day‐old seedlings and in mature, fully expanded leaves of 42‐day‐old soil‐grown plants. Analysis of fatty acid composition in phospholipids and galactolipids (c) of 15‐day‐old *pxa1* mutant plant lines (mean ± SD, *n* = 3; up and down arrowheads denote values significantly higher or lower, respectively, compared to the respective empty‐vector control at *P *=* *0.05).

**Figure S5** Analysis of polar lipids in *Arabidopsis cgi‐58* mutant plant lines. Content (a) and fatty acid composition (b) of polar lipids in 15‐day‐old seedlings and in mature, fully expanded leaves of 42‐day‐old soil‐grown plants (mean ± SD, *n* = 3; asterisks denote significant difference from respective empty‐vector control at *P* = 0.05; up and down arrowheads denote values significantly higher or lower, respectively, compared to the respective empty‐vector control at *P *=* *0.05).

**Figure S6** Phenotypes of *Arabidopsis cgi‐*58 mutant plant lines expressing tung *FADX* and/or *DGAT2*. Images of (a) 35‐day‐old and (b) 42‐day‐old soil‐grown plants. (c) Electrolyte leakage assay of mature, fully expanded leaves from 35‐day‐old plants (mean ± SD, *n* = 3).

**Figure S7** Properties of seeds derived from *Arabidopsis pxa1* mutant plant lines. (a) Images of mature, dry seeds (at 3.5× magnification. (b) Percentage of seed germination. (c) Seed oil content determined by NMR (mean ± SD, *n* = 5; asterisks denote values significantly different from respective empty‐vector control at *P *=* *0.05). (d) Fatty acid composition of seed oil (mean ± SD, *n* = 5; up and down arrowheads denote values significantly higher or lower, respectively, compared to the respective empty‐vector control at *P *=* *0.05).

**Figure S8** Properties of seeds from *Arabidopsis cgi‐58* mutant plant lines. (a) Images of mature, dry seeds (at 3.5× magnification). (b) Percentage of seed germination. (c) Seed oil content determined by NMR (mean ± SD, *n* = 5; asterisks denote values significantly different from respective empty‐vector control at *P *=* *0.05). (d) Fatty acid composition of seed oil (mean ± SD, *n* = 5; up and down arrowheads denote values significantly higher or lower, respectively, compared to the respective empty‐vector control at *P *=* *0.05).

**Figure S9** Distribution of ESA in lipids of *Arabidopsis* transgenic plant lines. (a) Content of ESA in neutral and polar lipids, plotted based on total mass amounts in fully expanded leaves of 42‐day‐old, soil‐grown plants of WT lines (mean ± SD, *n* = 3). (b) Percentage of ESA in neutral and polar lipids, plotted by setting the total mass amounts of ESA for each plant line in (a) to 100%. (c) Content of ESA in neutral and polar lipids, plotted based on total mass amounts for *pxa1* mutant plant lines (mean ± SD, *n* = 3). (d) Percentage of ESA in neutral and polar lipids, plotted by setting the total mass amounts of ESA for each plant line in (c) to 100%.

**Table S1** Summary of *Arabidopsis* transgenic lines and respective empty plasmid controls generated and analysed for the presence of α‐eleostearic acid (ESA) in total lipids extracted from 15‐day‐old seedlings. EV1 – empty vector 1 (also referred to as binary vector B9; see ‘Experimental procedures’ for additional details); EV2 – empty vector 2.

**Table S2** Oligonucleotide primer sequences used in qRT‐PCRs.
