# Supplementary figures and images for: Engineering the production of conjugated fatty acids in Arabidopsis thaliana leaves
Source: Plant Biotechnol J. 2017 Mar 15;15(8):1010–23. doi: 10.1111/pbi.12695 (PMC5506653; doi:10.1111/pbi.12695)

(a)

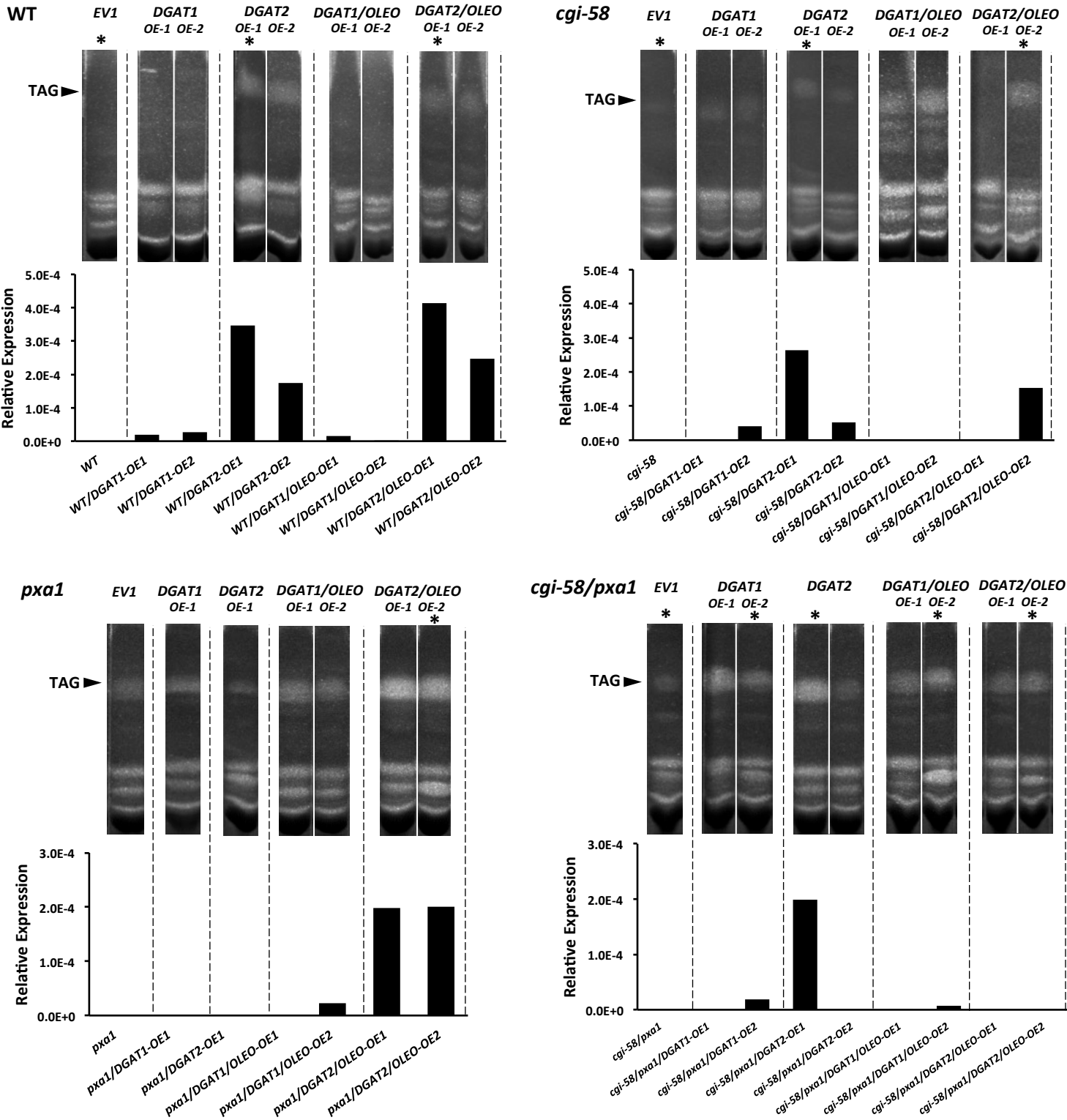

(b)

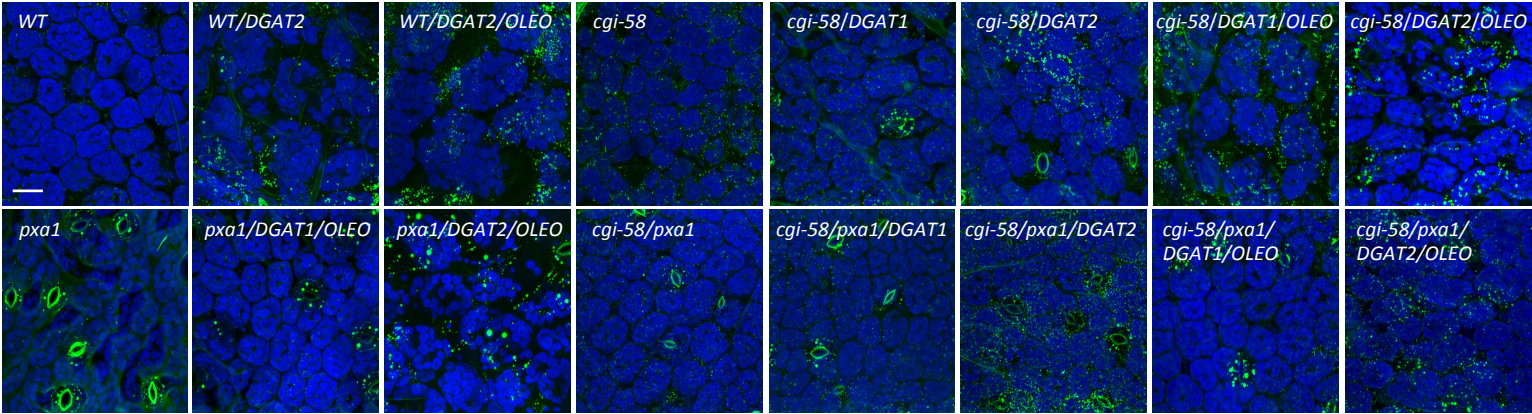

Supplement: Supplementary file 1 — Figure S1 Analysis of selected high‐leaf‐oil Arabidopsis transgenic lines. (a) TLC and gene expression analysis, with genotype/transgene combinations shown at the bottom of each bar graph. TLC analysis of lipids derived from mature, fully expanded leaves of 42‐day‐old plants are shown above the respective bar graphs showing qRT‐PCR analysis of tung DGAT1 or DGAT2 transcripts in 15‐day‐old seedlings relative to endogenous Arabidopsis 18S rRNA. Only the lanes for the top two TAG‐containing lines for each genotype/transgene combination are shown. The position of the TAG standard is shown to the left. EV1–Empty Vector 1. Asterisks denote plant lines selected for subsequent transformation with tung FADX or with EV2–empty vector 2. (b) Confocal fluorescence micrographs of mature, fully expanded leaves from 28‐day‐old Arabidopsis plant lines (as indicated by labels in panels) stained with BODIPY (lipid droplets appear green); chloroplast autofluorescence is coloured blue. Scale bar = 20 μm. [file PBI-15-1010-s005.pdf]

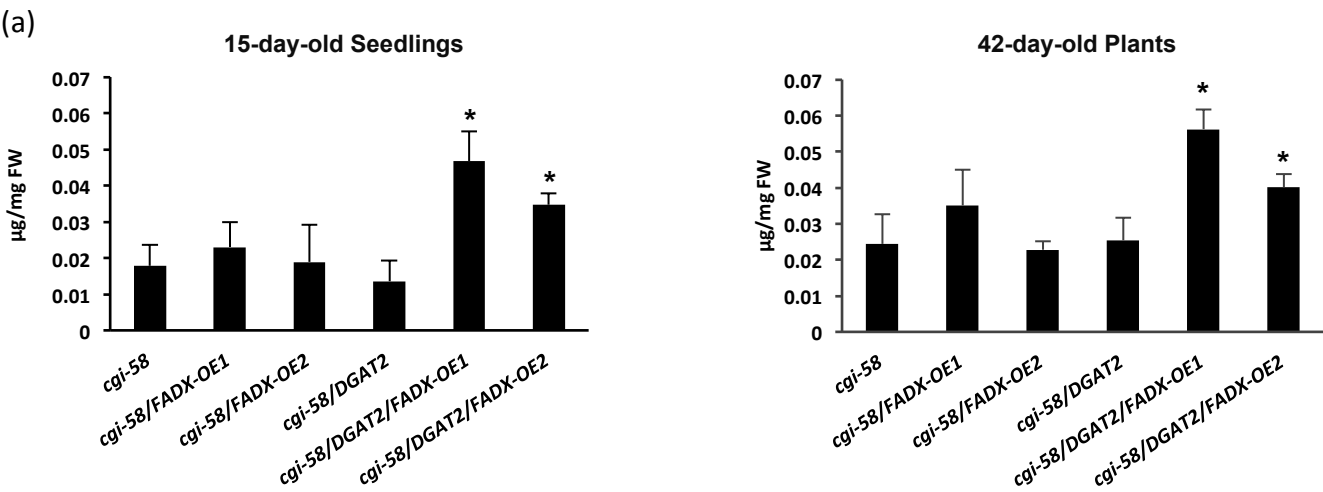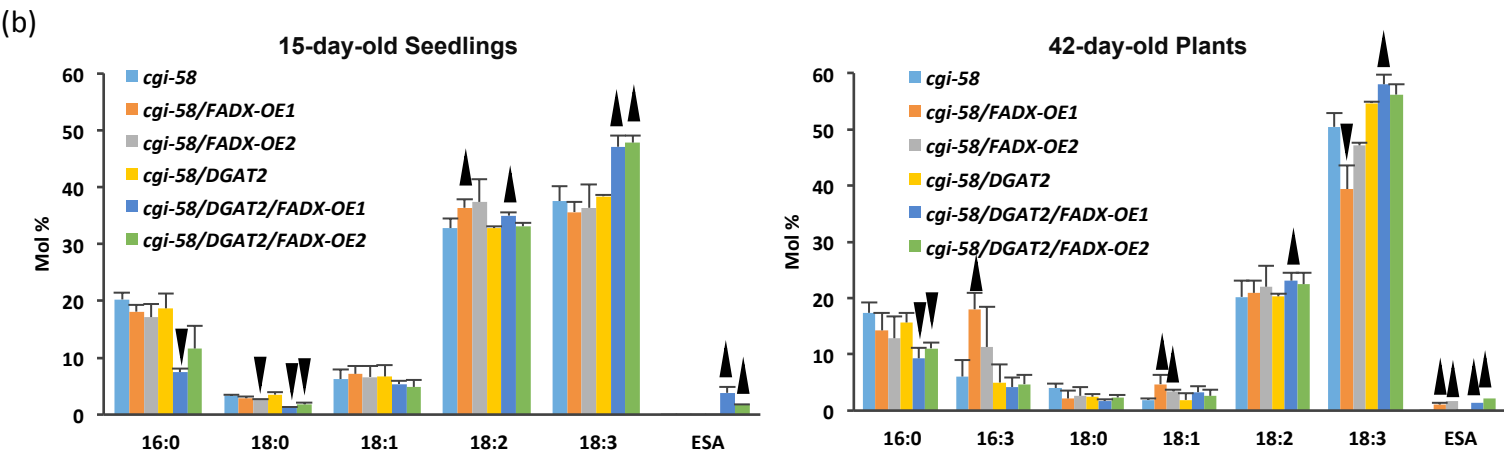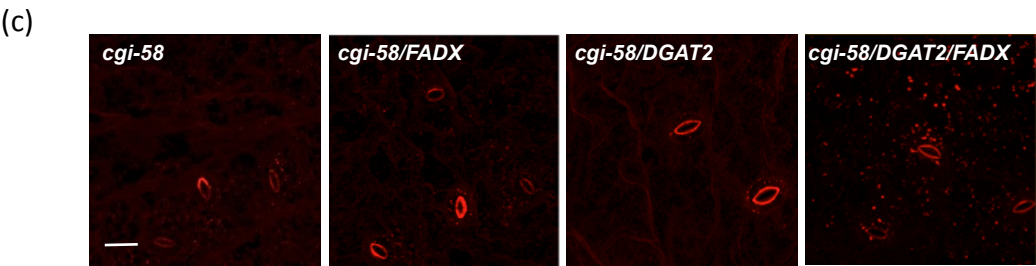

Supplement: Supplementary file 2 — Figure S2 Analysis of neutral lipids and lipid droplets in Arabidopsis cgi‐58 mutant plant lines. (a) Content of neutral lipids in 15‐day‐old seedlings and in mature, fully expanded leaves of 42‐day‐old, soil‐grown plants (mean ± SD, n = 3; asterisks denote significant difference from respective empty‐vector control at P = 0.05). (b) Fatty acid composition of neutral lipids in 15‐day‐old seedlings and in mature leaves of 42‐day‐old plants (mean ± SD, n = 3; up and down arrowheads denote values significantly higher or lower, respectively, compared to the respective empty‐vector control at P = 0.05). (c) Confocal fluorescence micrographs of Nile red‐stained lipid droplets in 15‐day‐old seedlings of cgi‐58 lines. Scale bar = 20 μm. [file PBI-15-1010-s009.pdf]

(a)

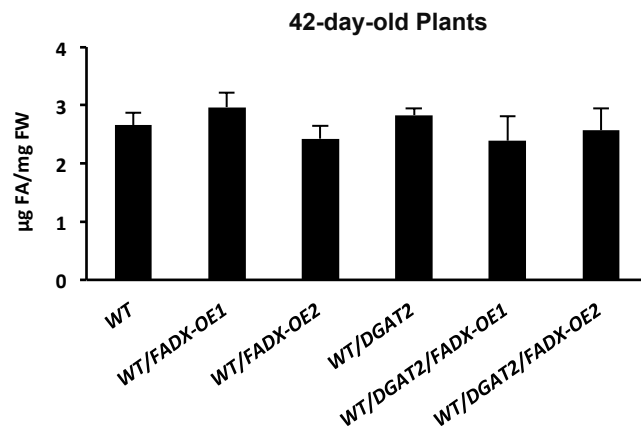

(b)

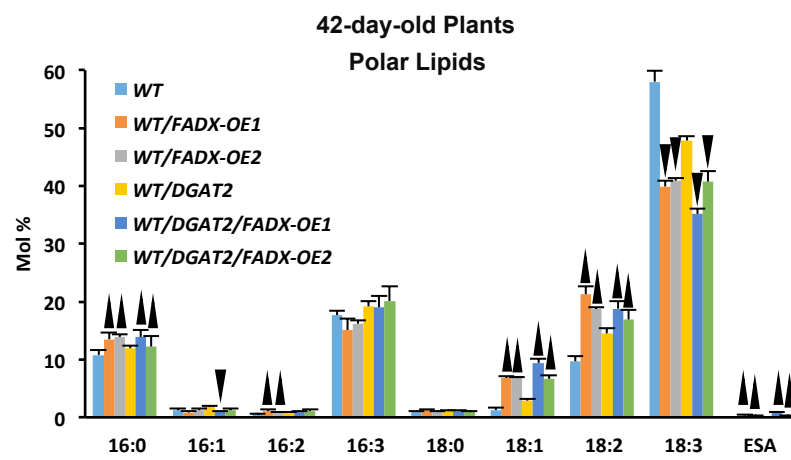

Supplement: Supplementary file 3 — Figure S3 Analysis of polar lipids in Arabidopsis WT plant lines. Content (a) and fatty acid composition (b) of polar lipids derived from mature, fully expanded leaves of 42‐day‐old, soil‐grown plants (mean ± SD, n = 3; up and down arrowheads denote values significantly higher or lower, respectively, compared to the respective empty‐vector control at P = 0.05). [file PBI-15-1010-s008.pdf]

(a)

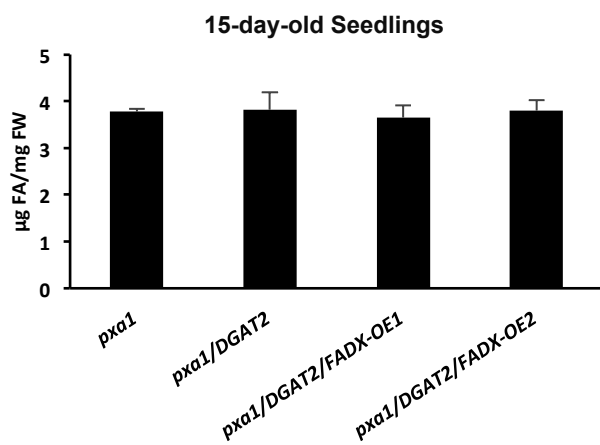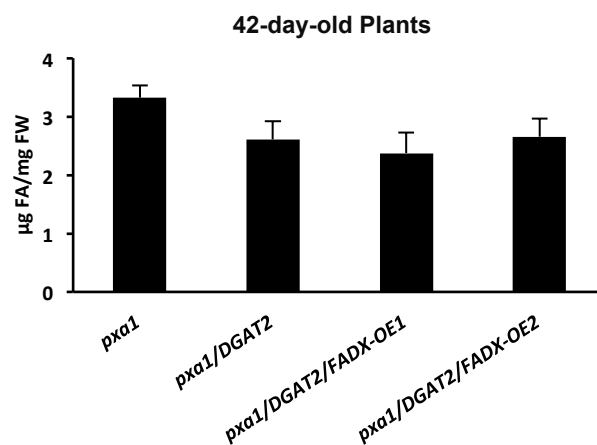

(b)

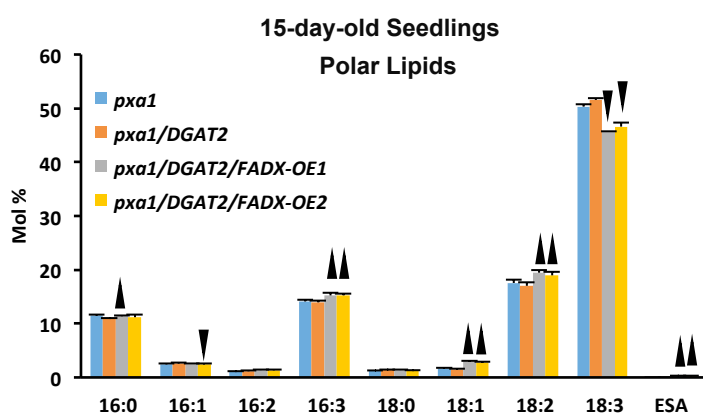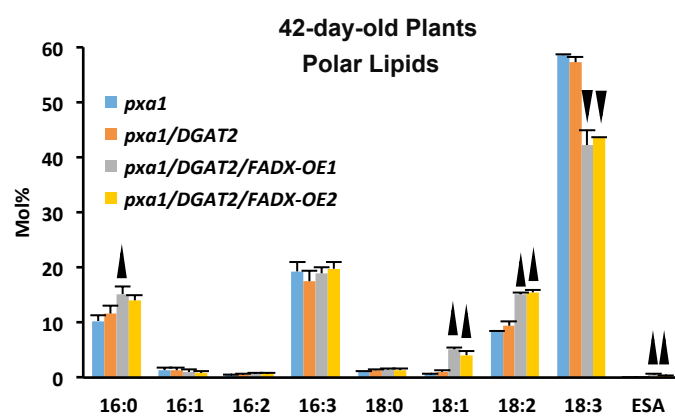

(c)

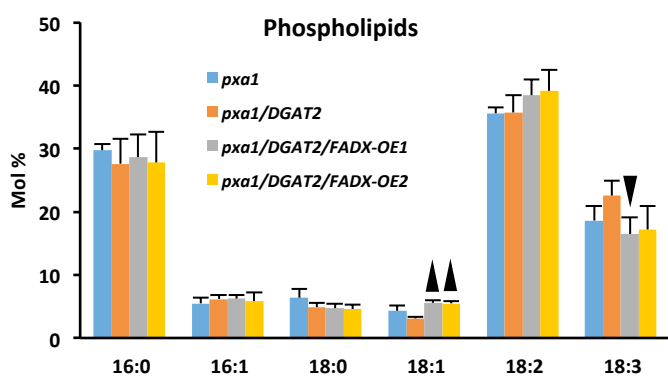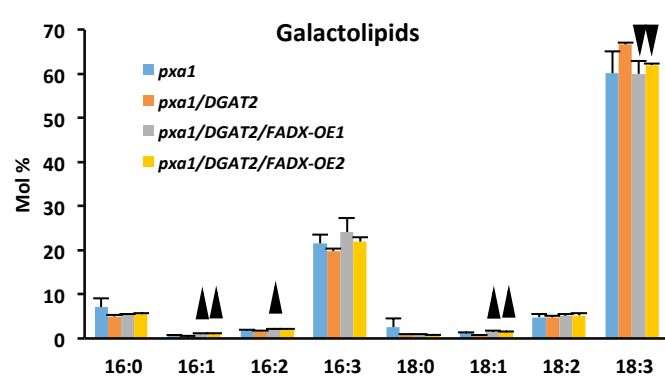

Supplement: Supplementary file 4 — Figure S4 Analysis of polar lipids in Arabidopsis pxa1 mutant plant lines. Content (a) and fatty acid composition (b) of polar lipids in 15‐day‐old seedlings and in mature, fully expanded leaves of 42‐day‐old soil‐grown plants. Analysis of fatty acid composition in phospholipids and galactolipids (c) of 15‐day‐old pxa1 mutant plant lines (mean ± SD, n = 3; up and down arrowheads denote values significantly higher or lower, respectively, compared to the respective empty‐vector control at P = 0.05). [file PBI-15-1010-s010.pdf]

(a)

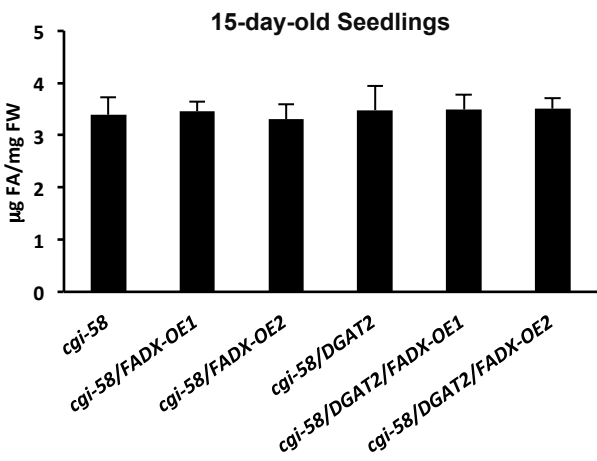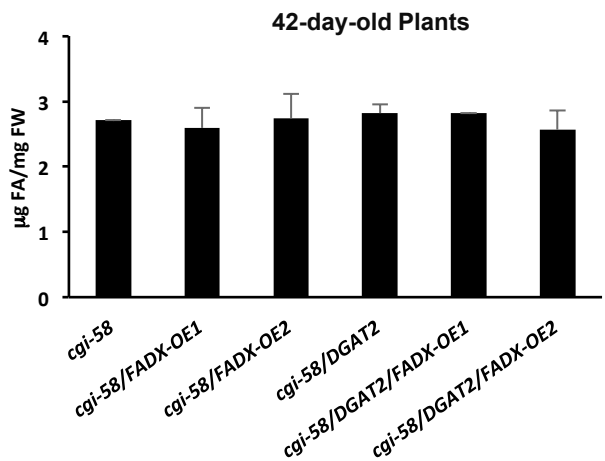

(b)

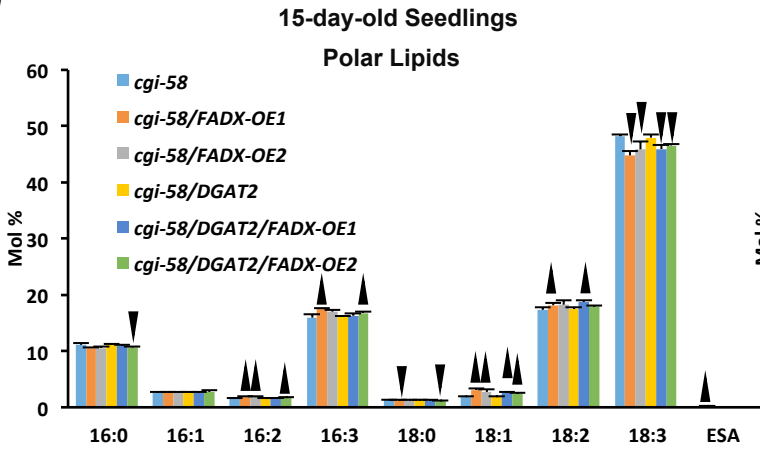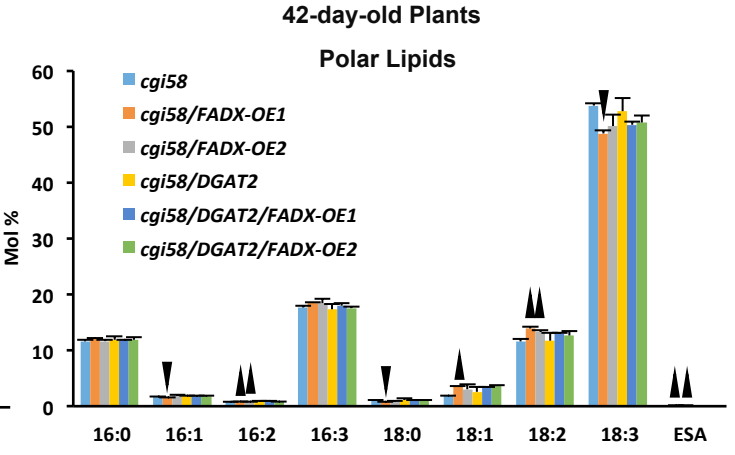

Supplement: Supplementary file 5 — Figure S5 Analysis of polar lipids in Arabidopsis cgi‐58 mutant plant lines. Content (a) and fatty acid composition (b) of polar lipids in 15‐day‐old seedlings and in mature, fully expanded leaves of 42‐day‐old soil‐grown plants (mean ± SD, n = 3; asterisks denote significant difference from respective empty‐vector control at P = 0.05; up and down arrowheads denote values significantly higher or lower, respectively, compared to the respective empty‐vector control at P = 0.05). [file PBI-15-1010-s001.pdf]

(a) *cgi-58/NV* *cgi-58/FADX-OE1* *cgi-58/FADX-OE2* *cgi-58/DGAT2/NV* *cgi-58/DGAT2/FADX-OE1* *cgi-58/DGAT2/FADX-OE2*

Day 35

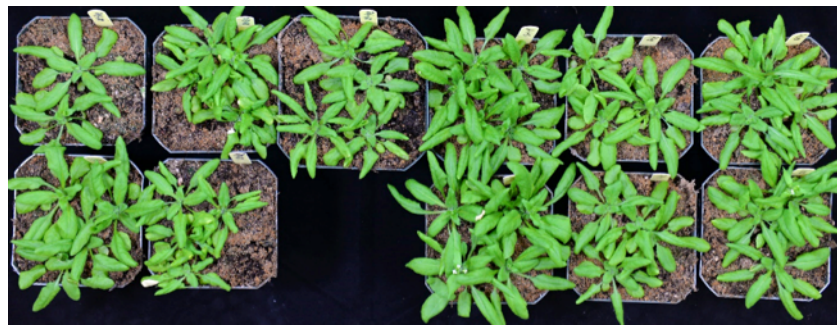

(b)

Day 42

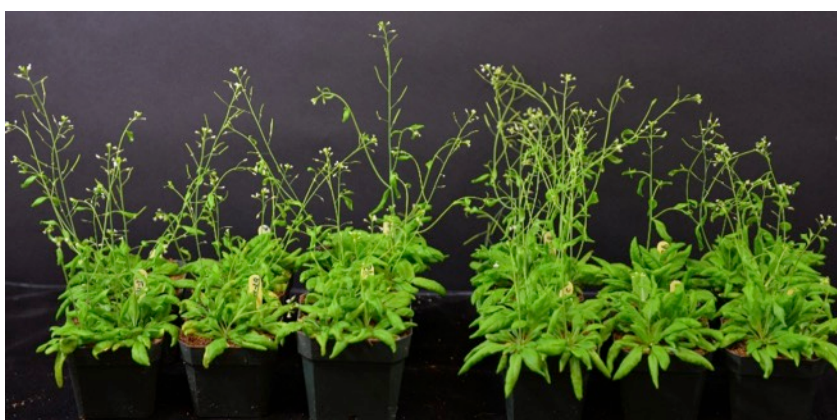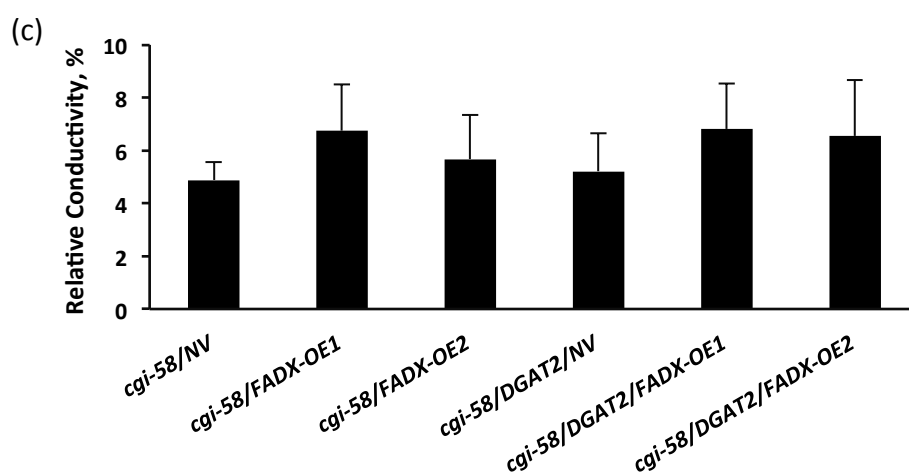

Supplement: Supplementary file 6 — Figure S6 Phenotypes of Arabidopsis cgi‐58 mutant plant lines expressing tung FADX and/or DGAT2. Images of (a) 35‐day‐old and (b) 42‐day‐old soil‐grown plants. (c) Electrolyte leakage assay of mature, fully expanded leaves from 35‐day‐old plants (mean ± SD, n = 3). [file PBI-15-1010-s002.pdf]

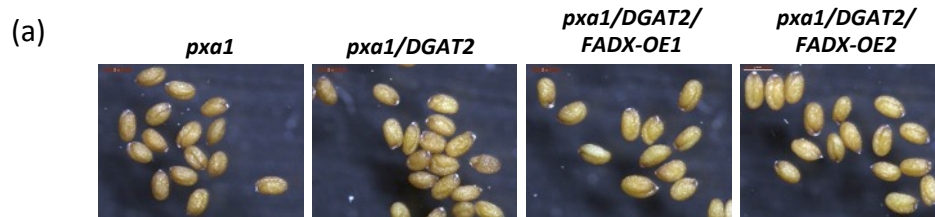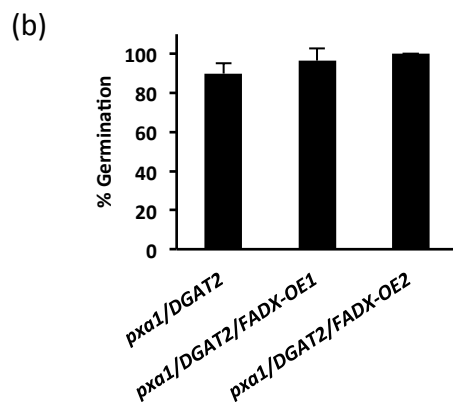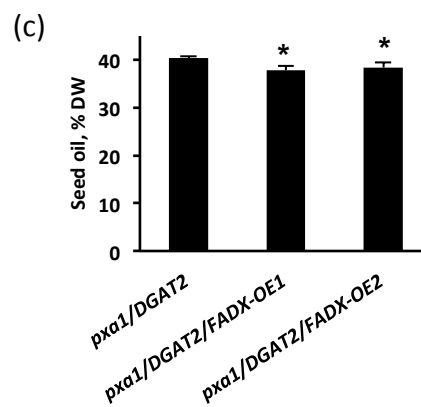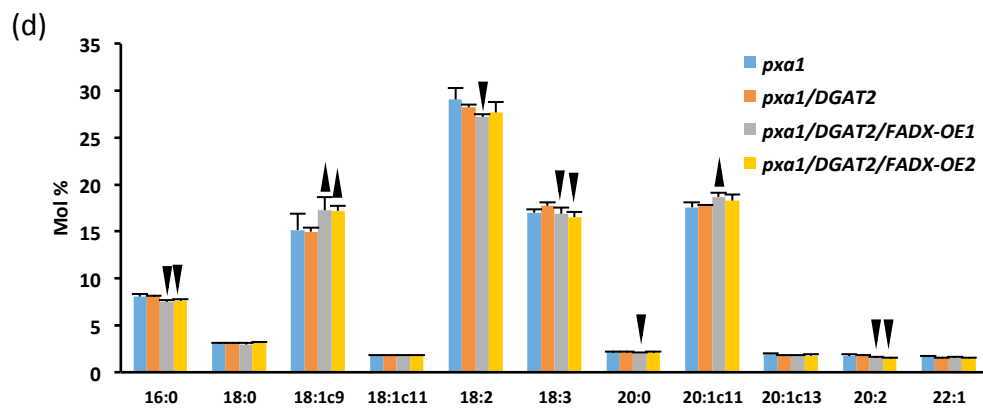

Supplement: Supplementary file 7 — Figure S7 Properties of seeds derived from Arabidopsis pxa1 mutant plant lines. (a) Images of mature, dry seeds (at 3.5× magnification. (b) Percentage of seed germination. (c) Seed oil content determined by NMR (mean ± SD, n = 5; asterisks denote values significantly different from respective empty‐vector control at P = 0.05). (d) Fatty acid composition of seed oil (mean ± SD, n = 5; up and down arrowheads denote values significantly higher or lower, respectively, compared to the respective empty‐vector control at P = 0.05). [file PBI-15-1010-s003.pdf]

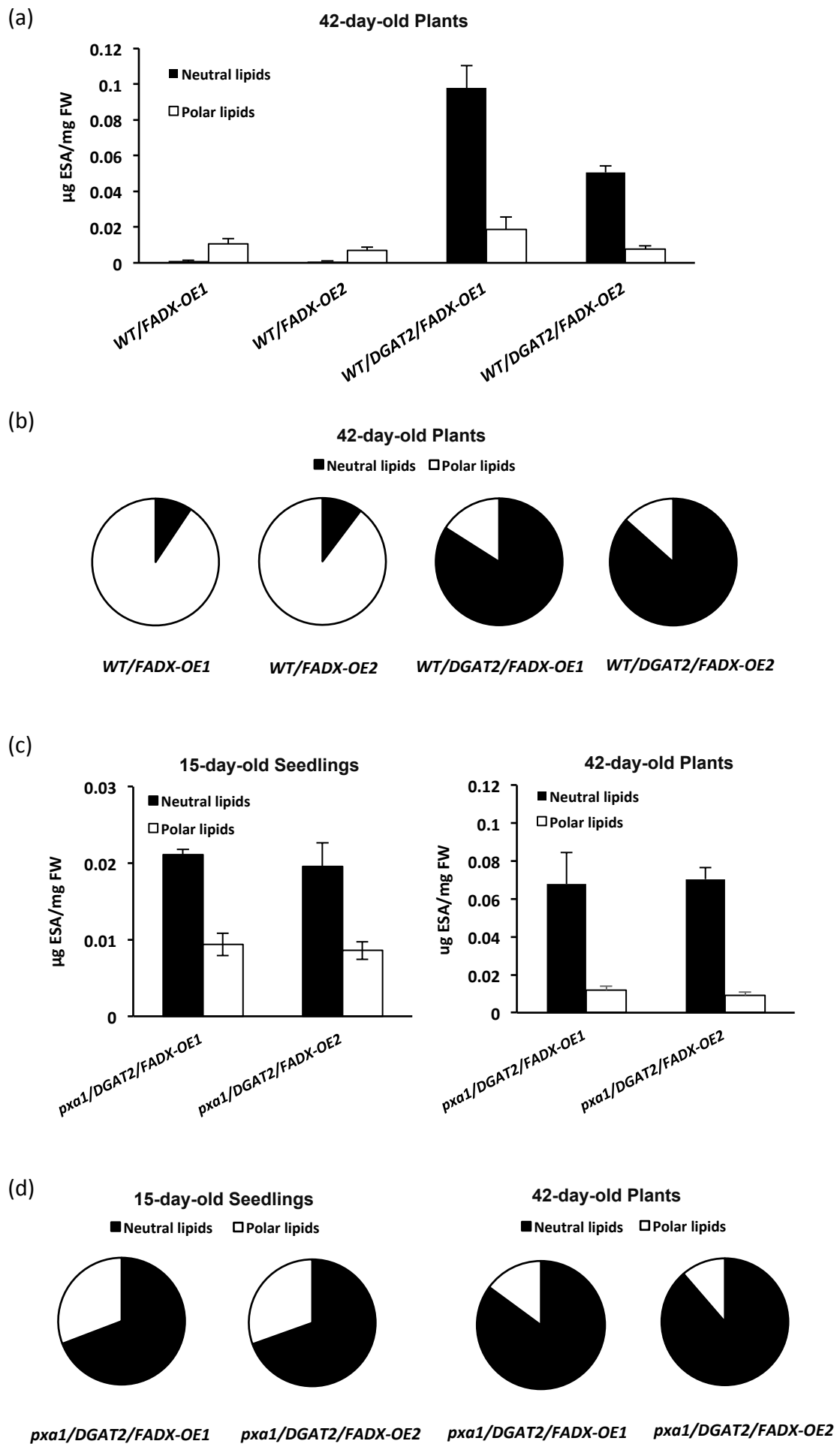

Supplement: Supplementary file 9 — Figure S9 Distribution of ESA in lipids of Arabidopsis transgenic plant lines. (a) Content of ESA in neutral and polar lipids, plotted based on total mass amounts in fully expanded leaves of 42‐day‐old, soil‐grown plants of WT lines (mean ± SD, n = 3). (b) Percentage of ESA in neutral and polar lipids, plotted by setting the total mass amounts of ESA for each plant line in (a) to 100%. (c) Content of ESA in neutral and polar lipids, plotted based on total mass amounts for pxa1 mutant plant lines (mean ± SD, n = 3). (d) Percentage of ESA in neutral and polar lipids, plotted by setting the total mass amounts of ESA for each plant line in (c) to 100%. [file PBI-15-1010-s011.pdf]
