# Supplementary material for: Engineering the production of conjugated fatty acids in Arabidopsis thaliana leaves
Source: Plant Biotechnol J. 2017 Mar 15;15(8):1010–23. doi: 10.1111/pbi.12695 (PMC5506653; doi:10.1111/pbi.12695)
Supplement: Supplementary file 10 — Table S1 Summary of Arabidopsis transgenic lines and respective empty plasmid controls generated and analysed for the presence of α‐eleostearic acid (ESA) in total lipids extracted from 15‐day‐old seedlings. EV1 – empty vector 1 (also referred to as binary vector B9; see ‘Experimental procedures’ for additional details); EV2 – empty vector 2. [file PBI-15-1010-s006.pdf]

Table S1 Summary of *Arabidopsis* transgenic lines and respective empty plasmid controls generated and analyzed for the presence of α-eleostearic acid (ESA) in total lipids extracted from 15-day-old seedlings. EV1 - empty vector 1 (also referred to as binary vector B9; see 'Experimental Procedures' for additional details); EV2 - empty vector 2.

Genetic makeup of the parental line	Genetic construct	# of T_2 lines harvested	# of T_2 lines used for progeny analysis	# of T_2 single-insertion lines identified	# of T_2 lines used for lipid analysis	# of T_2 lines with ESA (%ESA in total lipids)	# of T_3 lines used for lipid analyses
WT/EV1	EV2	16	5	3	1	0	1
WT/EV1	*FADX*	13	13	6	6	3 (0.25, 0.41, 0.69)	2
WT/*DGAT2*	EV2	15	5	4	1	0	1
WT/*DGAT2*	*FADX*	14	14	6	6	5 (2.87, 2.78, 1.76, 2.31, 2.65)	2
WT/*DGAT2*/*OLEO*	EV2	16	5	1	1	0	0
WT/*DGAT2*/*OLEO*	*FADX*	16	16	8	8	2 (0.43, 0.25)	0
cgi-58/EV1	EV2	16	5	3	1	0	1
cgi-58/EV1	*FADX*	15	15	7	6	2 (0.58, 0.32)	2
cgi-58/*DGAT2*	EV2	16	5	1	1	0	1
cgi-58/*DGAT2*	*FADX*	10	10	5	5	3 (0.61, 0.19, 0.91)	2
cgi-58/*DGAT2*/*OLEO*	EV2	15	5	3	1	0	0
cgi-58/*DGAT2*/*OLEO*	*FADX*	16	16	9	7	0	0
pxa1/*DGAT2*/*OLEO**	EV2	16	5	1	1	0	1
pxa1/*DGAT2*/*OLEO**	*FADX*	14	14	9	10	8 (0.42, 0.15, 0.31, 0.27, 0.25, 0.66, 0.66, 0.51)	2
cgi-58/*pxa1*/EV1	EV2	10	5	1	1	0	0
cgi-58/*pxa1*/EV1	*FADX*	3	3	1	1	0	0
cgi-58/*pxa1*/*DGAT1*	EV2	6	5	1	1	0	0
cgi-58/*pxa1*/*DGAT1*	*FADX*	8	8	5	5	0	0
cgi-58/*pxa1*/*DGAT2*	EV2	5	5	3	1	0	0
cgi-58/*pxa1*/*DGAT2*	*FADX*	4	4	2	2	0	0
cgi-58/*pxa1*/*DGAT1*/*OLEO*	EV2	7	5	1	1	0	0
cgi-58/*pxa1*/*DGAT1*/*OLEO*	*FADX*	2	2	1	2	1 (0.18)	0
cgi-58/*pxa1*/*DGAT2*/*OLEO*	EV2	8	5	2	1	0	0
cgi-58/*pxa1*/*DGAT2*/*OLEO*	*FADX*	8	8	6	5	0	0

* qRT-PCR analysis of T_3 transgenics derived from this line revealed that oleosin gene expression was negligible in comparison to transgenically-expressed tung *DGAT2* or endogenous *ACTIN8*, and, thus, the *pxa1*/*DGAT2*/*OLEO* line and subsequent *FADX* transgenics were renamed *pxa1*/*DGAT2* and *pxa1*/*DGAT2*/*FADX*, respectively.
