# Supplementary material for: Engineering the production of conjugated fatty acids in Arabidopsis thaliana leaves
Source: Plant Biotechnol J. 2017 Mar 15;15(8):1010–23. doi: 10.1111/pbi.12695 (PMC5506653; doi:10.1111/pbi.12695)
Supplement: Supplementary file 11 — Table S2 Oligonucleotide primer sequences used in qRT‐PCRs. [file PBI-15-1010-s007.pdf]

**Table S2** Oligonucleotide primer sequences used in qRT-PCR.

| Gene/Transcript | Primer sequences (5' to 3') |
|---|---|
| *VfDGAT1* | *Fp GGGGTGGCCTTATTAATTACTTTCTTC*<br>*Rp TGAGCTTCTGAACTTGTTTTGAAGG* |
| *VfDGAT2* | *Fp GAAACCTGATGGAGAGTTGTTCATG*<br>*Rp GATGCAATAAACTCTCTCTGTACTTCC* |
| *VfOLEOSINII* | *FpCCTTTGTTCTTGGCCTTGCT*<br>*Fp TGTCCCACATAACCAGCCAT* |
| *VfFADX* | *Fp ATCACATCCTTGCAGCACAG*<br>*Rp CCAATATCGGCTTGATTGCT* |
| *AtACX4* | *Fp TCTGGCTGTCTCACGTGTAA*<br>*Rp TCTGACCCGTCTCATACAGC* |
| *AtLPCAT1* | *Fp GAACGGAAAGAAAGCGGGTT*<br>*Rp ATGAAACCGACGGCTGAGTA* |
| *AtACTIN8* | *Fp AGTGGTCGTACAACCGGTATTGT*<br>*Rp GAGGATAGCATGTGGAACTGAGAA* |
| *At18S* | *Fp TCATTACTCCGATCCCGAAG*<br>*Rp ACGCTCCTGGTCTTAATTGG* |
