# Supplementary material for: Engineering the production of conjugated fatty acids in Arabidopsis thaliana leaves
Source: Plant Biotechnol J. 2017 Mar 15;15(8):1010–23. doi: 10.1111/pbi.12695 (PMC5506653; doi:10.1111/pbi.12695)
Supplement: Supplementary file 8 — Figure S8 Properties of seeds from Arabidopsis cgi‐58 mutant plant lines. (a) Images of mature, dry seeds (at 3.5× magnification). (b) Percentage of seed germination. (c) Seed oil content determined by NMR (mean ± SD, n = 5; asterisks denote values significantly different from respective empty‐vector control at P = 0.05). (d) Fatty acid composition of seed oil (mean ± SD, n = 5; up and down arrowheads denote values significantly higher or lower, respectively, compared to the respective empty‐vector control at P = 0.05). [file PBI-15-1010-s004.pdf]

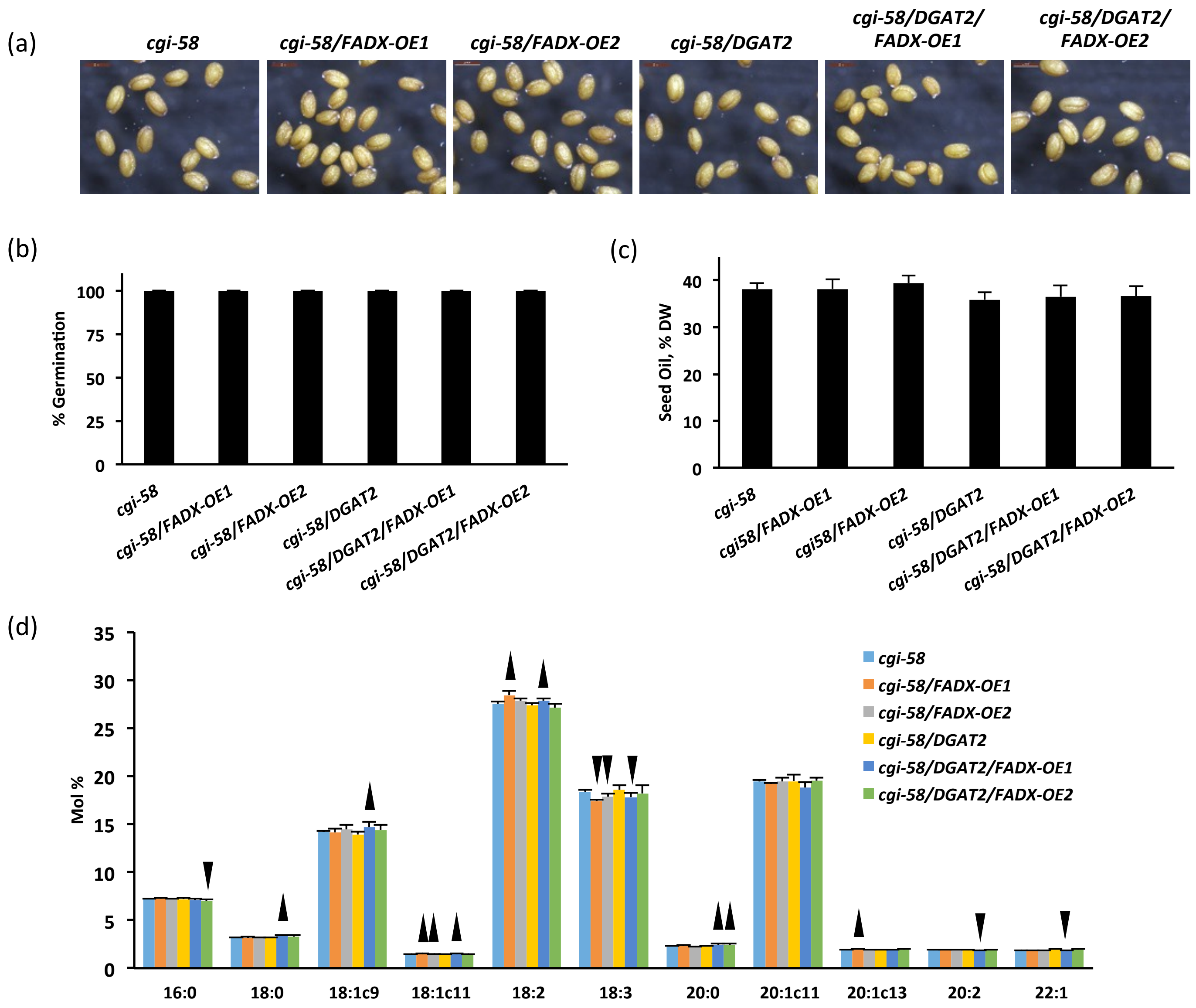
(a)
cgi-58
cgi-58/FADX-OE1
cgi-58/FADX-OE2
cgi-58/DGAT2
cgi-58/DGAT2/ FADX-OE1
cgi-58/DGAT2/ FADX-OE2
(b)
% Germination
100
75
50
25
0
cgi-58
cgi-58/FADX-OE1
cgi-58/FADX-OE2
cgi-58/DGAT2
cgi-58/DGAT2/FADX-OE1
cgi-58/DGAT2/FADX-OE2
(c)
Seed Oil, % DW
40
30
20
10
0
cgi-58
cgi58/FADX-OE1
cgi58/FADX-OE2
cgi-58/DGAT2
cgi-58/DGAT2/FADX-OE1
cgi-58/DGAT2/FADX-OE2
(d)
Mol %
35
30
25
20
15
10
5
0
16:0
18:0
18:1c9
18:1c11
18:2
18:3
20:0
20:1c11
20:1c13
20:2
22:1
cgi-58
cgi-58/FADX-OE1
cgi-58/FADX-OE2
cgi-58/DGAT2
cgi-58/DGAT2/FADX-OE1
cgi-58/DGAT2/FADX-OE2
